# Supplementary figures and images for: Yes-Associated Protein Drives Helicobacter pylori–Induced Metaplastic Changes in Gastric Epithelium
Source: Cell Mol Gastroenterol Hepatol. 2026 May 21;20(9):101814. doi: 10.1016/j.jcmgh.2026.101814 (PMC13324660; doi:10.1016/j.jcmgh.2026.101814)

Figure 1

L

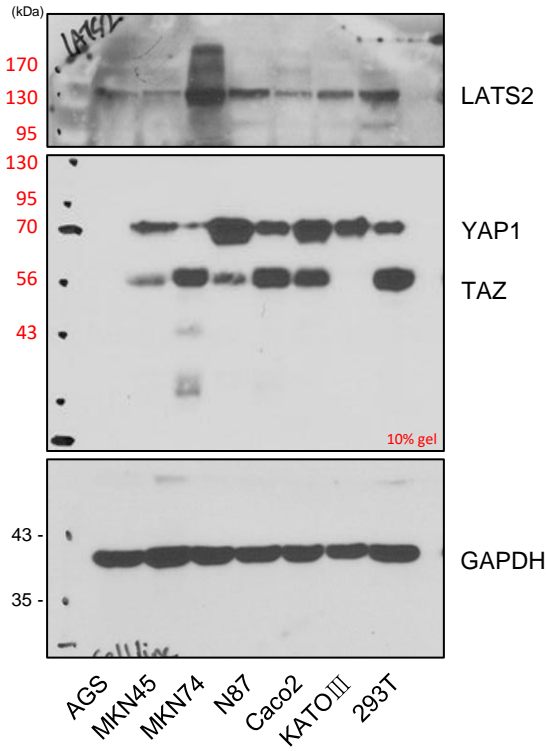

M

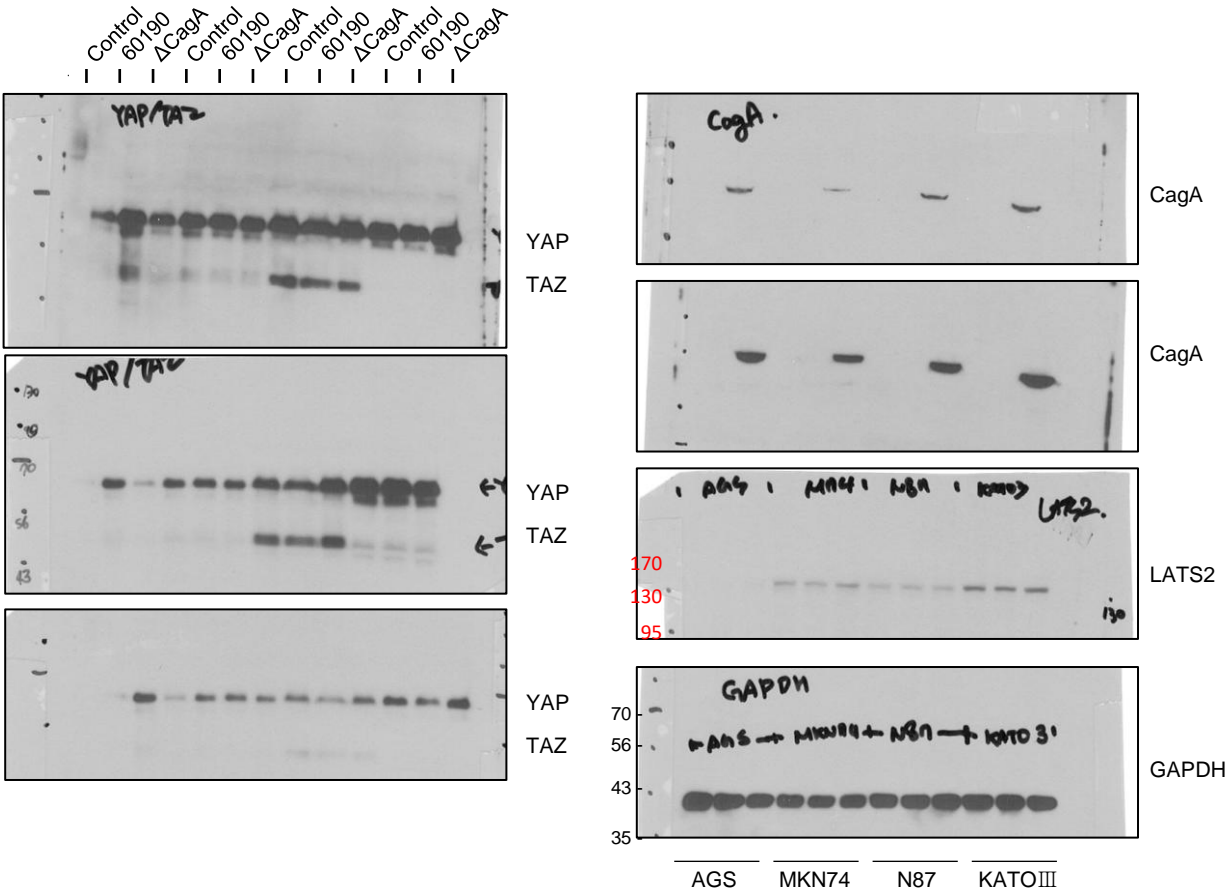

Figure 2

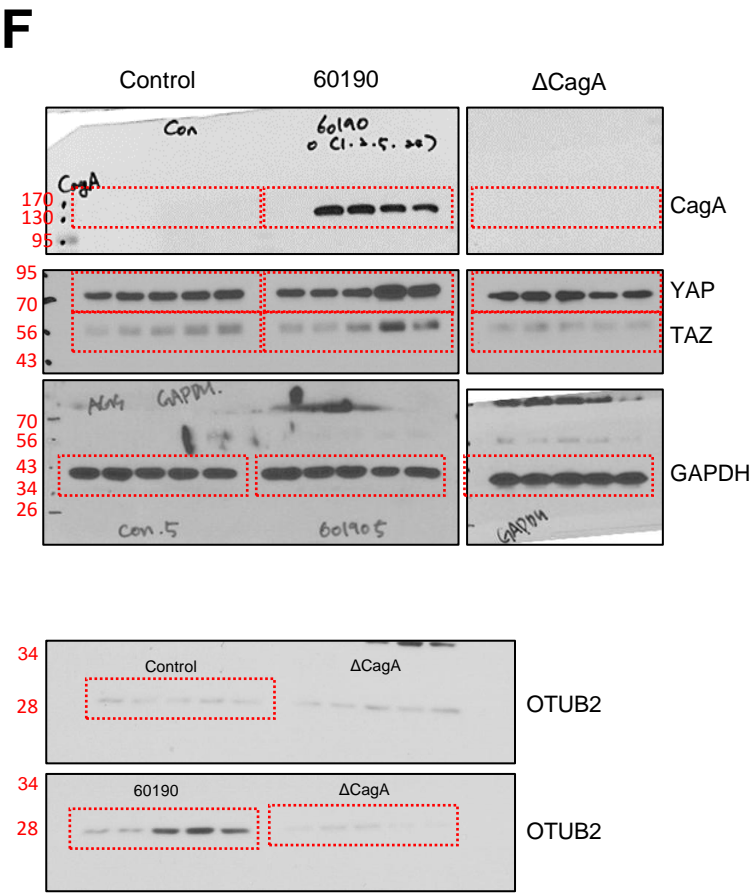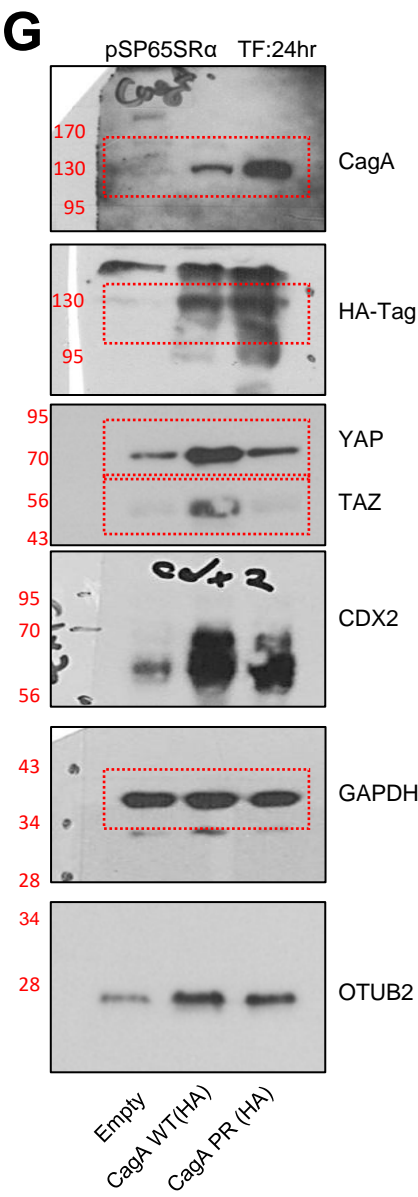

Figure 3

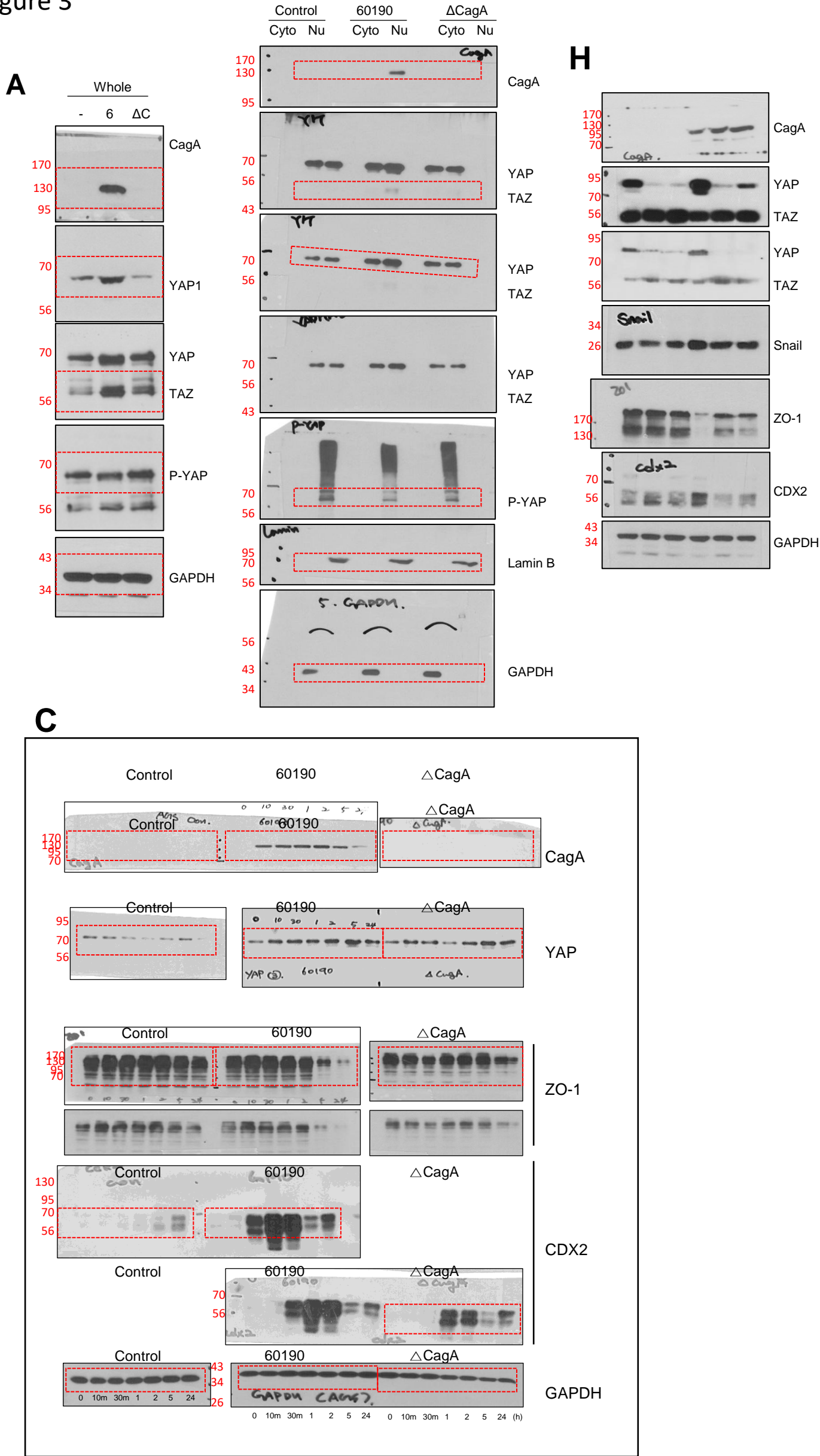

Figure 4

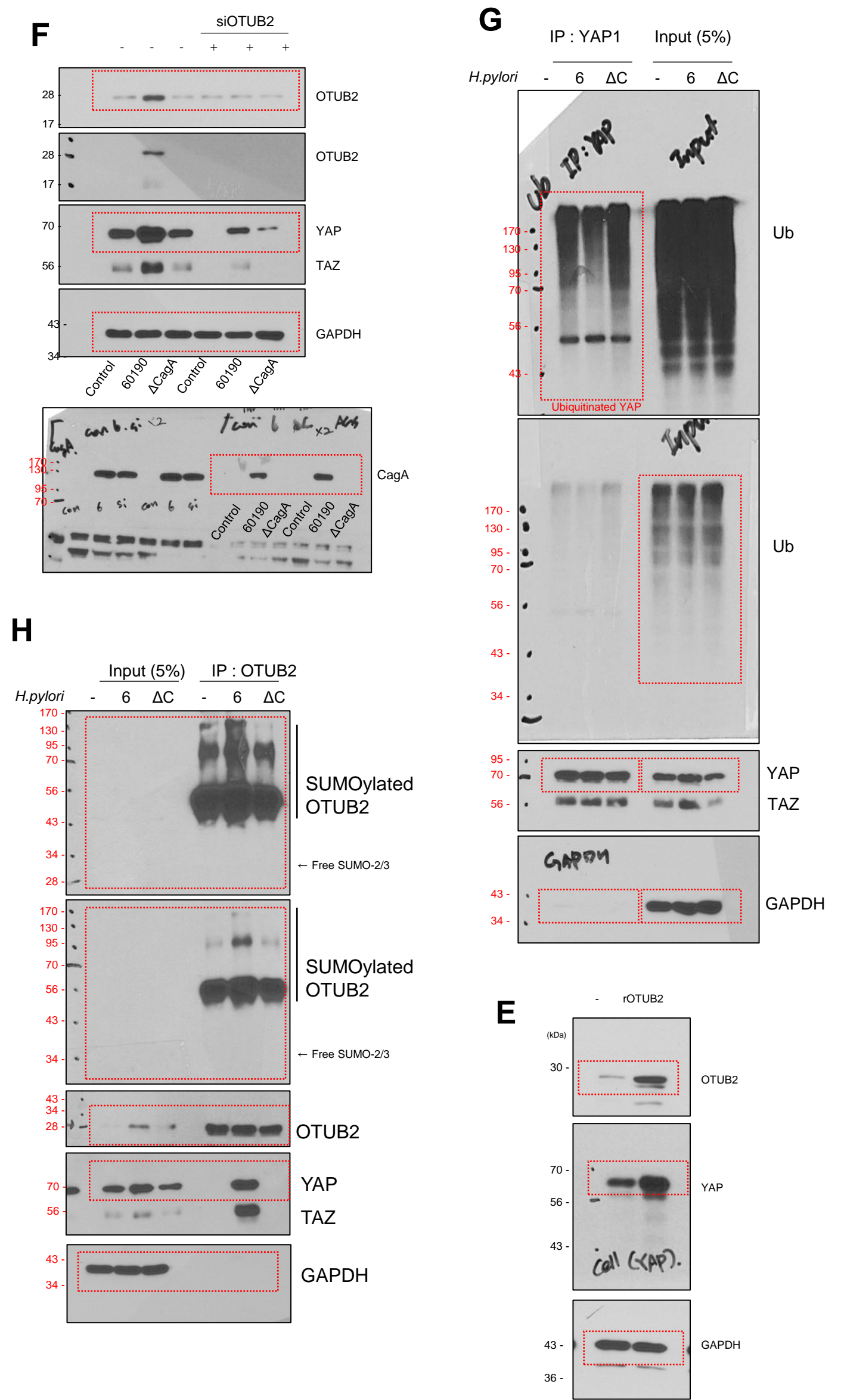

Figure 4

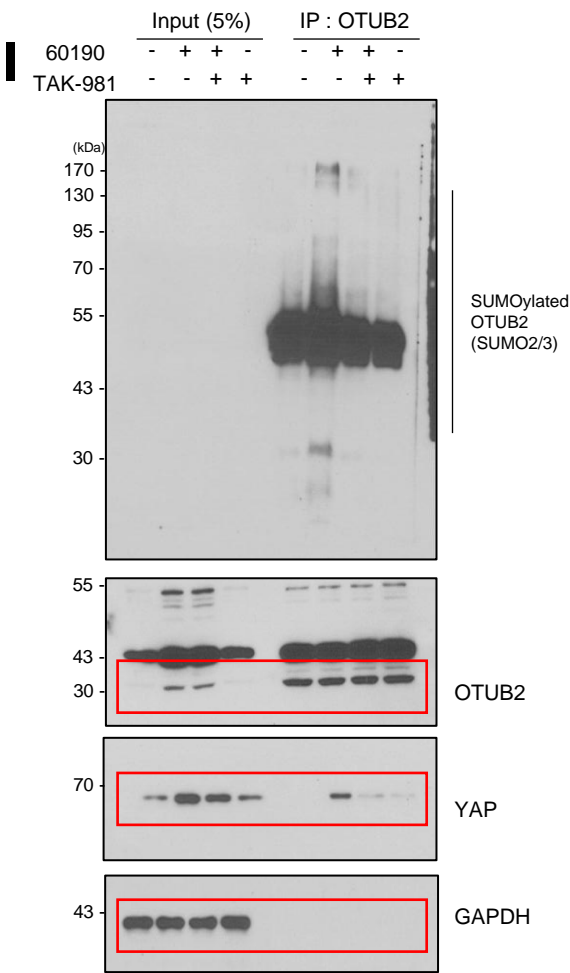

Supplement: Supplementary Figures [file mmc1.pdf]
