# Supplementary material for: Yes-Associated Protein Drives Helicobacter pylori–Induced Metaplastic Changes in Gastric Epithelium
Source: Cell Mol Gastroenterol Hepatol. 2026 May 21;20(9):101814. doi: 10.1016/j.jcmgh.2026.101814 (PMC13324660; doi:10.1016/j.jcmgh.2026.101814)
Supplement: Extended PDF [file mmc3.pdf]

cmgh
CELLULAR AND MOLECULAR GASTROENTEROLOGY AND HEPATOLOGY

ORIGINAL RESEARCH

# Yes-Associated Protein Drives *Helicobacter pylori*–Induced Metaplastic Changes in Gastric Epithelium

Byeong Min Yu,[1] So Dam Lee,[1] Bo Ram Hwang,[1] Haengdueng Jeong,[2,4] Jiseon Kim,[2] Seokyoung Hwang,[3] Minsoo Noh,[3] Ki Taek Nam,[2] and Yong Chan Lee[1]

[1]*Department of Internal Medicine, Institute of Gastroenterology, Yonsei University College of Medicine, Seoul, Republic of Korea;* [2]*Department of Biomedical Science, Graduate School of Medical Science, Brain Korea 21 Project, Yonsei University College of Medicine, Seoul, Republic of Korea;* [3]*College of Pharmacy, Seoul National University, Seoul, Republic of Korea; and* [4]*Department of Molecular and Life Science, Hanyang University, Ansan, Republic of Korea*

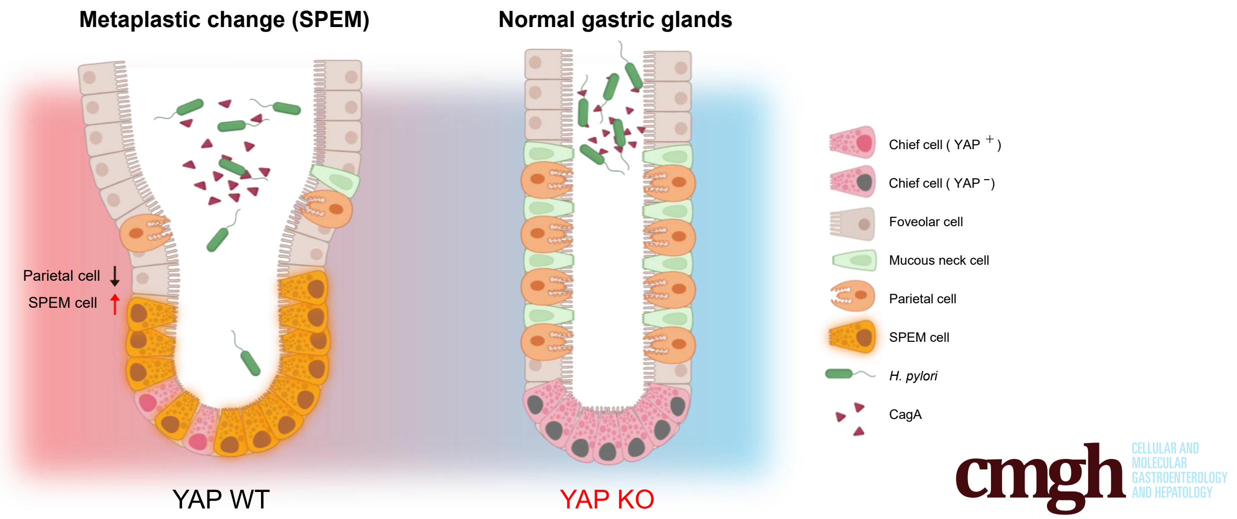


## SUMMARY

*Helicobacter pylori* promotes gastric metaplastic transformation through CagA-dependent activation of YAP signaling. Genetic and pharmacologic studies identify OTUB2 as an upstream regulator of YAP and a potential target for preventing metaplastic progression.

## WHAT YOU NEED TO KNOW

**Background:** *Helicobacter pylori* infection induces gastric metaplasia, but upstream regulators remain unclear. Yes-associated protein signaling has emerged as a key pathway linking bacterial virulence factors to epithelial reprogramming.

**Impact:** This study identifies OTU deubiquitinase, ubiquitin aldehyde binding 2–mediated yes-associated protein stabilization as a central driver of *H pylori*–induced gastric metaplasia, linking cytotoxin-associated gene A signaling to chief cell reprogramming and disease progression.

**Future Directions:** Future studies should validate yes-associated protein activity and downstream targets in human gastric metaplastic and neoplastic tissues and explore pharmacologic inhibition to prevent metaplasia progression.

**BACKGROUND & AIMS:** *Helicobactor pylori* infection triggers a spectrum of metaplastic changes in the gastric epithelium; however, the upstream regulatory mechanisms remain unclear. In human gastric epithelial models, *H pylori* cytotoxin-associated gene A–dependent signaling stabilized and activated yes-associated protein, thereby promoting metaplastic reprogramming of the gastric epithelium.

**METHODS:** Human gastric tissue specimens were obtained from patients at Severance Hospital, Yonsei University College

of Medicine. Human gastric epithelial cell lines (AGS, MKN74, N87, KATO III) and 8- to 10-week-old C57BL/6J mice were used. Transcriptomic analysis, immunoprecipitation, histologic and immunofluorescence staining, and molecular assays (Western blotting and quantitative reverse-transcription polymerase chain reaction) were performed to assess yes-associated protein signaling and metaplastic marker expression.

**RESULTS:** Genetic and pharmacologic modulation of yes-associated protein and OTU deubiquitinase, ubiquitin aldehyde binding 2 demonstrated that these metaplastic marker alterations are yes-associated protein–dependent. In vivo, comparative infection with *H pylori* with or without cytotoxin-associated gene A revealed significantly increased spasmolytic polypeptide–expressing metaplasia markers from chief cells (Griffonia simplicifolia II lectin/gastric intrinsic factor, CD44 variant 9), parietal cell loss, and an expanded proliferative region in the presence of cytotoxin-associated gene A. *Helicobacter felis* infection was used to broaden the dynamic range of in vivo pathologic outcomes. Manipulating yes-associated protein specifically in chief cells with a muscle, intestine, stomach expression 1 driver verified both the induction and suppression of these lesions, and the administration of recombinant OTU deubiquitinase, ubiquitin aldehyde binding 2 and treatment with selective inhibitors enhanced and reduced metaplastic signaling, respectively.

**CONCLUSIONS:** Yes-associated protein acts as a central regulator of *H pylori*–induced metaplastic transformation by reprogramming gastric epithelial and chief cell lineages. OTU deubiquitinase, ubiquitin aldehyde binding 2 serves as an upstream modulator that reinforces yes-associated protein stability and signaling, highlighting yes-associated protein as a potential therapeutic target for preventing metaplastic progression. *(Cell Mol Gastroenterol Hepatol 2026;20:101814; https://doi.org/10.1016/j.jcmgh.2026.101814)*

*Keywords:* Gastric Metaplasia; *H pylori*; Spasmolytic Polypeptide-Expressing Metaplasia (SPEM); YAP.

*Helicobacter pylori* infection is a canonical driver of multistep gastric pathology, progressing from chronic gastritis through metaplasia and dysplasia to gastric carcinoma.[1–5] The major *H pylori* virulence factor cytotoxin-associated gene A (CagA) perturbs host signaling pathways, disrupts epithelial junctional integrity, and promotes a tumor-permissive inflammatory microenvironment.[6–11] Although these early epithelial alterations are widely recognized as initiating events in gastric carcinogenesis, the molecular mechanism through which bacterial virulence signals are translated into epithelial reprogramming remain incompletely defined.[12,13]

Yes-associated protein (YAP) is a transcriptional coactivator that integrates cues from cell adhesion, polarity, and mechanical signaling to engage TEA domain transcription factor (TEAD)-dependent transcriptional programs.[14–17] In the stomach, YAP activation and metaplastic remodeling are frequently observed in gastric mucosa from patients with chronic *H pylori* infection and are considered early events preceding neoplastic transformation.[18–22] Emerging studies have positioned YAP as a central regulator of epithelial plasticity and injury-induced cell fate reprogramming.[22,23] However, in gastric epithelial cells, the upstream mechanisms through which CagA-dependent signals converge on YAP activation have not been clearly established.[12,13,18,20]

OTU deubiquitinase, ubiquitin aldehyde binding 2 (OTUB2) is a deubiquitinase capable of stabilizing YAP through the modulation of ubiquitin signaling, raising the possibility that it acts as an upstream mediator linking microbial cues to YAP activation.[24,25] Nevertheless, how OTUB2 activity is regulated during *H pylori* infection and whether post-translational mechanisms contribute to YAP stabilization in this context remain largely unexplored. In particular, whether small ubiquitin-like modifier (SUMO)ylation, a post-translational modification known to regulate protein function and localization, is engaged by bacterial virulence signals to modulate YAP stability has not been determined.[26,27]

Gastric metaplasia represents a spectrum of epithelial reprogramming states rather than a single pathologic entity.[28] Human epithelial models enable precise interrogation of epithelial-intrinsic transcriptional changes, including intestinal-type programs typified by caudal type homeobox (CDX) 2 induction.[29–32] In contrast, in vivo systems preserve the composite tissue microenvironment and are required to capture tissue-level remodeling, such as spasmolytic polypeptide–expressing metaplasia (SPEM) arising from chief cells.[33–37] Given species-specific differences in gastric lineage markers, integrated analyses across multiple markers and at single-cell resolution are critical for accurate interpretation of metaplastic changes.[38,39] Therefore, understanding how YAP signaling integrates microbial stimuli with epithelial plasticity across these

*Abbreviations used in this paper:* **ATP4B, ATPase transporting subunit $H^+/K^+$ $\beta$; CagA, cytotoxin-associated gene A; CD44v9, CD44 variant 9; CDX, caudal type homeobox; CXCL8, chemokine C-X-C motif ligand 8; DEG, differentially expressed gene; ELISA, enzyme-linked immunosorbent assay; GAPDH, glyceraldehyde-3-phosphate dehydrogenase; GIF, gastric intrinsic factor; GKN, gastrokine; GO, Gene Ontology; GSEA, gene set enrichment analysis; GSII, Griffonia simplicifolia II lectin; H&E, hematoxylin and eosin; IF, immunofluorescence; IHC, immunohistochemistry; IL-8, interleukin 8; IM, intestinal metaplasia; IP, immunoprecipitation; ISH, in situ hybridization; KO, knockout; LATS2, large tumor suppressor kinase 2; MOI, multiplicity of infection; mRNA, messenger RNA; MUC, mucin; OTUB2, OTU deubiquitinase, ubiquitin aldehyde binding 2; PBS, phosphate-buffered saline; PGA, pepsinogen; PGC, pepsinogen C; rOTUB2, recombinant OTUB2; RT-PCR, reverse-transcription polymerase chain reaction; SDS, sodium dodecyl sulfate; shRNA, short-hairpin RNA; siRNA, small interfering RNA; SPEM, spasmolytic polypeptide-expressing metaplasia; SUMO, small ubiquitin-like modifier; TAZ, transcriptional coactivator with PDZ-binding motif; TEAD, TEA domain transcription factor; TFF, trefoil factor; WB, Western blot; WFDC2, WAP 4-disulfide core domain 2; WT, wild-type; YAP, yes-associated protein; ZO-1, zonula occludens-1.**

**Most current article**

**© 2026 The Author(s). Published by Elsevier Inc. on behalf of American Gastroenterological Association Institute. This is an open access article under the CC BY license (http://creativecommons.org/licenses/by/4.0/).**
**2352-345X**
**https://doi.org/10.1016/j.jcmgh.2026.101814**

interconnected metaplastic programs is central to elucidating the early mechanisms of gastric disease progression.[18,19,21,22]

In this study, we investigated whether *H pylori* CagA activates YAP signaling through OTUB2 and whether this pathway drives epithelial junctional disruption and metaplastic reprogramming. Using complementary human epithelial models and in vivo genetic approaches, we identified a CagA–OTUB2–YAP signaling axis that links microbial infection to epithelial remodeling and established YAP as a central regulator of metaplastic transformation in gastric epithelium during infection.

# Results

## Yes-Activated Protein Activation in Human Specimens, Single-Cell Data, and Cell Lines

Immunofluorescence (IF) analysis of human gastric specimens representing chronic superficial gastritis, intestinal metaplasia (IM), intestinal-type gastric cancer, and diffuse-type gastric cancer revealed a stepwise increase in YAP signaling with disease progression (Figure 1*A*). In contrast, the expression of the tight-junction protein zonula occludens-1 (ZO-1) progressively decreased across disease stages (Figure 1*A*), indicating that YAP activation accompanies the weakening of epithelial junctional integrity during the progression of gastric lesions. Consistently, the analysis of a public cohort using gene expression profiling interactive analysis demonstrated significantly higher YAP messenger RNA (mRNA) expression in tumor tissues, compared with that in matched adjacent normal tissues (Figure 1*B*).

To further define YAP expression at single-cell resolution, we integrated and reanalyzed 48,670 single-cell transcriptomes derived from nonmalignant and malignant human gastric tissues. After quality control and batch correction, cell populations were visualized using Uniform Manifold Approximation and Projection (Figure 1*C*). Mapping of cells by lesion stage revealed distinct shifts in cellular composition between non-neoplastic and tumor tissues (Figure 1*D*). Unsupervised clustering of the single-cell RNA sequencing dataset resolved 10 major cell clusters. Cluster identities were then inferred by analyzing cluster-enriched differentially expressed genes (DEGs) in conjunction with established canonical marker genes. On this basis, the clusters were annotated as endothelial cells (VWF and CD34), enteroendocrine cells (CHGA and CHGB), fibroblasts (LUM and PDGFRA), gland mucous cells (mucin [MUC] 6 and trefoil factor [TFF] 2), IM-associated epithelial cells (TFF3, CDX1, and CDX2), chief cells (pepsinogen [PGA] 4 and PGA5), myofibroblasts (ACTA2 and TAGLN), pit mucous cells (MUC5AC, TFF1, gastrokine [GKN] 1, and GKN2), parietal cells (ATPase transporting subunit $H^+/K^+$ $\beta$ [ATP4B] and CBLIF), and cancer cells (CEACAM5 and CEACAM6). These annotations were further supported by dot plot visualization of representative marker genes across clusters (Figure 1*E*), allowing integrated assessment of marker expression intensity and detection frequencies. Because gland mucous and metaplastic cell programs are known to partially overlap, cell identities were assigned on the basis of combined canonical marker expression and overall transcriptional features rather than by any single marker alone.[38] Accordingly, the gland mucous cell cluster may include cells with pyloric metaplasia–like features.[36] Visualization of YAP expression across cell types showed that YAP was broadly expressed across multiple epithelial lineages of the gastric epithelium (Figure 1*F*). Notably, chief cells derived from cancer tissues exhibited significantly higher YAP expression, compared with chief cells from noncancerous tissues (Figure 1*G*). Next, we performed unsupervised subclustering of chief cells and identified SPEM marker–negative and SPEM marker–positive subpopulations based on the expression of established SPEM-associated markers (Figure 1*H*). The SPEM marker–positive population was characterized by elevated expression of SPEM-associated markers (APQ5 and WAP 4-disulfide core domain 2 [WFDC2]), reduced expression of canonical chief cell markers (PGA4 and PGA5), and higher YAP expression (Figure 1*I* and *K*). Consistently, Uniform Manifold Approximation and Projection visualization revealed that aquaporin-5– and WFDC2-expressing cells were enriched along a metaplastic continuum within a subset of chief cells (Figure 1*J*), supporting the existence of heterogeneity within the chief cell compartment. Collectively, these findings indicate a gradual expansion of metaplastic programs during gastric lesion development and suggest that YAP expression is associated with the emergence of SPEM-like features within the chief cell lineage.

Additionally, we assessed the basal expression of core Hippo pathway components, including YAP, transcriptional coactivator with PDZ-binding motif (TAZ), and large tumor suppressor kinase 2 (LATS2), across a panel of human gastric cancer cell lines (Figure 1*L*). Cells exposed to *H pylori* strain 60,190 ($CagA^+$) for 5 hours showed a marked increase in YAP level, compared with that in uninfected controls and cells infected with the ΔCagA strain (Figure 1*M* and *N*), indicating CagA-dependent activation of YAP signaling. Based on these findings, subsequent in vitro experiments were performed using AGS cells infected with *H pylori* 60,190 ($CagA^+$) to elucidate the mechanism.

## Cytotoxin-Associated Gene A–. Positive H pylori Induces Alterations in Hippo Signaling, K63-Linked Deubiquitination, and Inflammatory Gene Expression in Gastric Cancer Cells

RNA-seq analysis revealed marked changes in Hippo signaling–associated genes (Gene Ontology [GO]:0035329) in AGS cells infected with *H pylori* 60,190 ($CagA^+$) (Figure 2*A*). Notably, the transcript levels of YAP, WWTR1 (TAZ), LATS2, SAV1, STK4, and TEAD1 were higher in the *H pylori* 60,190 than in uninfected controls and ΔCagA-infected cells. Gene set enrichment analysis (GSEA) further showed significant positive enrichment of YAP signaling-related signatures in *H pylori* 60,190-infected cells, compared with both uninfected controls and ΔCagA-infected cells (Figure 2*B*).

The K63-linked deubiquitination gene set displayed a higher enrichment score in *H pylori* 60,190–infected cells in both comparisons (control vs 60,190 and 60,190 vs ΔCagA), and OTUB2 was concomitantly upregulated at the transcript level under the same condition (Figure 2*C*).

C-X-C chemokine receptor chemokine receptor binding gene sets were positively enriched after *H pylori* 60,190 infection, relative to both uninfected controls and ΔCagA-infected cells, and chemokine C-X-C motif ligand 8 (CXCL8; interleukin-8 [IL-8]) expression increased (Figure 2*D*).

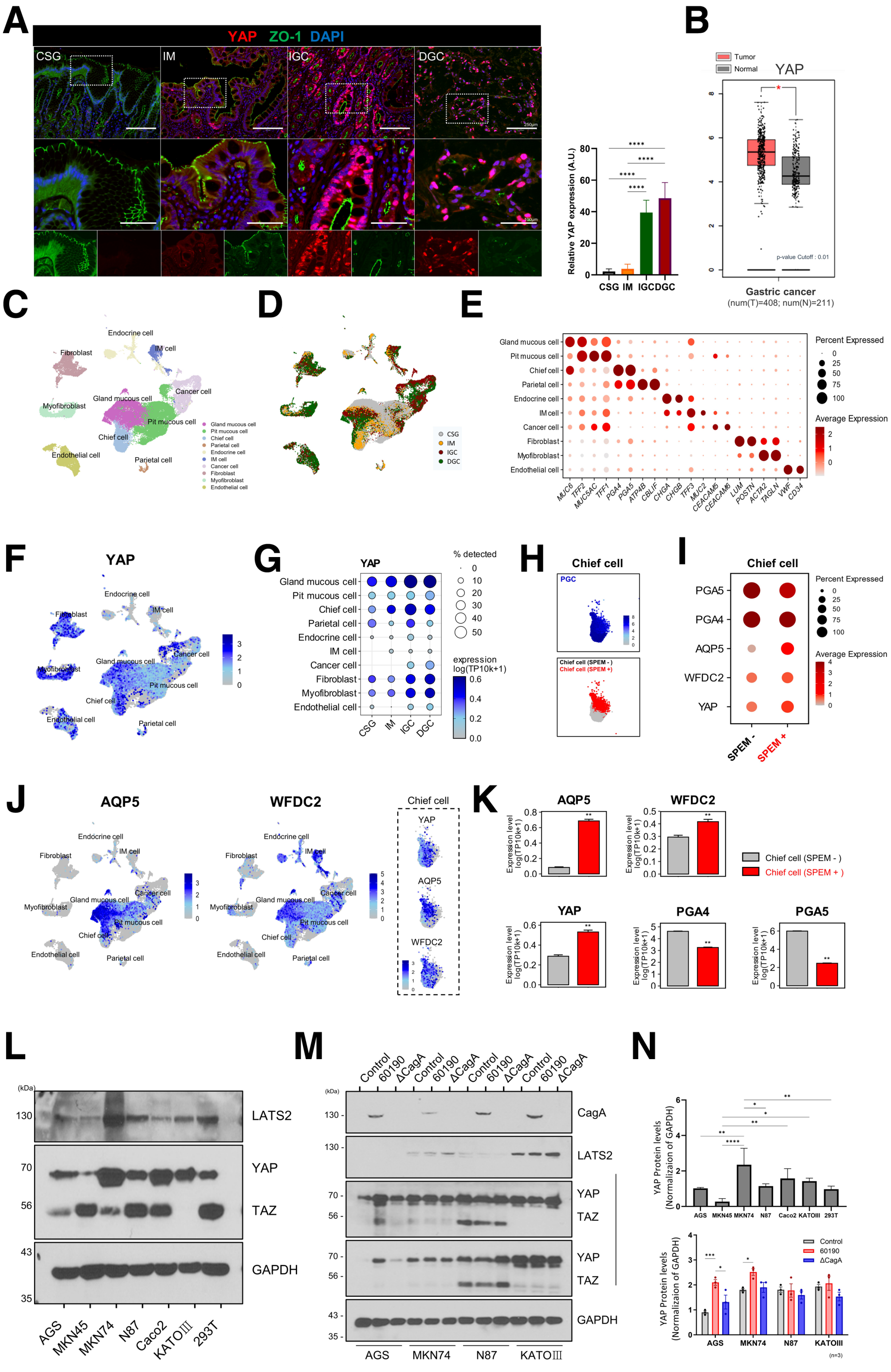

Consistent with the transcriptomic data, enzyme-linked immunosorbent assay (ELISA) at 8 hours postinfection showed significantly higher IL-8 secretion in *H pylori* 60,190–infected cells than in uninfected or ΔCagA-infected cells (Figure 2*E*). Time-course immunoblotting revealed the induction of YAP and TAZ proteins, with YAP peaking at approximately 5 hours postinfection (Figure 2*F*). Moreover, plasmid-mediated CagA overexpression (wild-type [WT] and phosphorylation-resistant mutant) increased the levels of YAP, TAZ, and the deubiquitinase OTUB2 (Figure 2*G*).

Collectively, these findings indicate that CagA-dependent signaling converges on the Hippo–YAP signaling, ubiquitin regulatory circuits, and inflammatory responses, motivating mechanistic interrogation of the OTUB2-regulated YAP signaling as an upstream node.

## Linking Yes-Associated Protein Nuclear Translocation to Tight-Junction Loss and Intestinal-Type Metaplastic Change

To determine whether *H pylori*–induced YAP activation involves nuclear translocation, we performed nuclear–cytoplasmic fractionation in AGS cells. Nuclear YAP levels were markedly increased at 5 hours postinfection with *H pylori* 60,190 ($CagA^{+}$), compared with control and ΔCagA strain (Figure 3*A*). Consistent with this finding, quantitative fluorescence imaging revealed a prominent accumulation of YAP within the nuclei following 5 hours of *H pylori* 60,190 infection (Figure 3*B*).

To assess whether nuclear YAP accumulation correlates with epithelial barrier integrity and intestinal differentiation, we evaluated the expression of ZO-1 and CDX2. Immunoblotting and IF analyses revealed a decrease in ZO-1, a tight-junction protein, and a concomitant increase in CDX2, a representative marker of intestinal metaplasia (Figure 3*C* and *D*). These changes were consistently observed at the transcript level, as reverse-transcription polymerase chain reaction (RT-PCR) showed reduced ZO-1 mRNA and elevated CDX2 mRNA, following *H pylori* 60,190 infection (Figure 3*E*).

Next, we examined whether YAP activation results in functional transcriptional output. TEAD luciferase reporter activity increased following *H pylori* 60,190 infection. However, this induction was abolished in YAP-depleted cells (shYAP#1/#2), indicating that transcriptional activation is YAP-dependent (Figure 3*F*). Similarly, siYAP-treated cells exhibited reduced IL-8 production (Figure 3*G*), and YAP depletion blunted the infection-induced decrease in ZO-1 expression and increase in CDX2 expression (Figure 3*H* and *I*). These findings support a causal role for YAP in regulating junctional integrity and metaplastic gene programs.

Based on these in vitro observations, we evaluated whether YAP contributes to proliferative potential in vivo. In an AGS xenograft model, tumors derived from YAP-depleted cells (shYAP#1 or shYAP#2-mediated) exhibited reduced tumor growth. Bioluminescence imaging revealed significantly lower radiant efficiency in tumors formed by YAP-depleted cells, compared with pLKO.1 control tumors, indicating that YAP depletion suppresses tumor growth in vivo (Figure 3*J*).

To investigate the structural role of YAP in epithelial morphogenesis, YAP expression was modulated by electroporation in normal gastric organoids. YAP suppression markedly impaired organoid formation and growth, suggesting that an appropriate level of YAP activity is required

**Figure 1. Expression of YAP in human gastric lesions and cancer cell lines.** (*A*) Representative IF staining of YAP (*red*) and ZO-1 (*green*) in human gastric lesions: CSG, IM, IGC, and DGC. Scale bars, 250 and 100 $\mu$m. (*B*) GEPIA comparing YAP mRNA in tumors (n = 408) vs matched adjacent normal tissues (n = 211). Mann–Whitney *U* test; **P* < .05. (*C*) UMAP visualization of 48,670 single cells derived from paired nonmalignant and malignant gastric tissues after quality control and batch correction. Ten major clusters identified via unsupervised clustering were annotated on the basis of the expression of canonical markers. (*D*) UMAP distribution of single cells stratified by lesion stage. Cells are colored according to lesion stage to visualize stage-specific distributions across CSG, IM, IGC, and DGC. (*E*) Dot plot showing the expression of canonical marker genes across annotated cell types. *Dot size* indicates the percentage of cells expressing each gene, and *color intensity* indicates the average expression level. (*F*) UMAP feature plot showing YAP expression across cell populations. (*G*) Dot plot showing YAP expression across annotated cell types, stratified by lesion stage. *Color* indicates average expression level, and *dot size* indicates the fraction of cells expressing YAP. (*H*) Subclustering of chief cells based on SPEM-associated markers. Chief cells were classified into SPEM marker–negative and SPEM marker–positive populations according to the expression of AQP5 and WFDC2. *Red* indicates SPEM marker–positive chief cells, and *gray* indicates SPEM marker–negative chief cells. (*I*) Dot plot showing differential expression of canonical chief cell markers (PGA4 and PGA5) and SPEM-associated markers (AQP5 and WFDC2), along with YAP expression, between SPEM marker–negative and SPEM marker–positive chief cell populations. (*J*) UMAP feature plots showing the expression of SPEM-associated markers AQP5 and WFDC2. The inset highlights their spatial enrichment within a subset of chief cells, along with YAP expression. (*K*) Quantitative analysis of gene expression in SPEM marker–negative and SPEM marker–positive chief cells, confirming increased YAP, AQP5, and WFDC2 expression and decreased PGA4 and PGA5 expression in the SPEM marker-positive population. Data are presented as log (TP10k + 1); mean ± SEM. (*L*) Representative WB showing basal protein levels of Hippo pathway components (YAP, TAZ, and LATS2) in gastric cancer cell lines (AGS, N87, KATO III, MKN45, and MKN74), a colorectal line (Caco-2), and a renal epithelial line (293T) (n = 3). (*M*) WB analysis of YAP, TAZ, LATS2, and CagA expression after incubating cells with control (uninfected), *H pylori* 60,190 (CagA+), or ΔCagA strains (n = 3) for 5 hours. (*N*) Densitometry of YAP bands normalized to GAPDH (for *L* and *M*). Statistics: Data are presented as mean ± SEM. Multi-group comparisons used 1-way or 2-way ANOVA with Tukey's post hoc test. **P* < .05; ***P* < .01; ****P* < .001; *****P* < .0001. APQ5, aquaporin-5; CSG, chronic superficial gastritis; DGC, diffuse-type gastric cancer; GEPIA, gene expression profiling interactive analysis; IGC, intestinal-type gastric cancer; SEM, standard error of the mean; UMAP, Uniform Manifold Approximation and Projection.

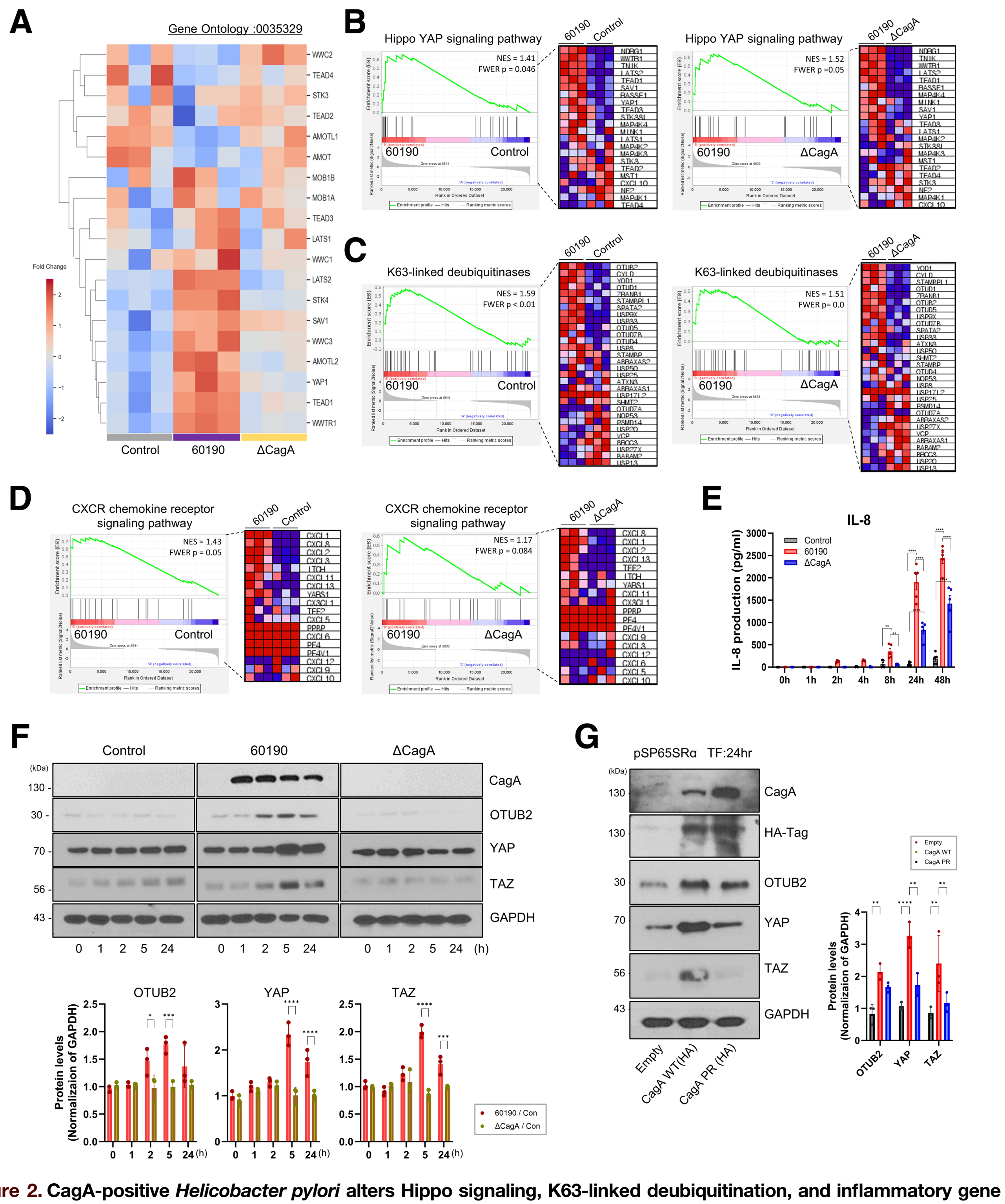


**Figure 2. CagA-positive *Helicobacter pylori* alters Hippo signaling, K63-linked deubiquitination, and inflammatory gene expression in gastric cancer cells.** (*A*) Heatmap of genes in the Hippo pathway GO term (GO: 0035329) in AGS cells infected with *H pylori* 60,190 (CagA+) or ΔCagA for 5 hours. (*B*) GSEA plot for YAP signaling signatures comparing Control vs *H pylori* 60,190 (CagA+) and *H pylori* 60,190 (CagA+) vs ΔCagA-infected AGS cells. (*C*) GSEA plot for K63-linked deubiquitination comparing control vs *H pylori* 60,190 (CagA$^+$) and *H pylori* 60,190 (CagA$^+$) vs ΔCagA-infected AGS cells. (*D*) GSEA plot showing positive enrichment of the CXCR chemokine receptor binding gene set comparing control vs *H pylori* 60,190 (CagA$^+$) and *H pylori* 60,190 (CagA$^+$) vs ΔCagA-infected AGS cells. (*E*) ELISA of IL-8 production in AGS cells infected with 60,190 or ΔCagA (n = 4 per group). (*F*) Representative WB showing time-course of YAP and TAZ after infection with 60,190 or ΔCagA (n = 3). (*G*) Representative WB showing the induction of YAP/TAZ and OTUB2 after the overexpression of CagA WT or the EPIYA-PR mutant (n = 3). Statistics: Data are presented as mean ± SEM. Group comparisons used 2-way ANOVA with Tukey's post hoc test. *$P < .05$; **$P < .01$; ***$P < .001$; ****$P < .0001$. CXCR, C-X-C chemokine receptor; PR, phosphorylation-resistant; SEM, standard error of the mean.

for the maintenance of epithelial architecture. In contrast, YAP overexpression induced nuclear stratification and the formation of multiple lumens, resulting in an intestinalized morphology. This phenotype closely resembled that of patient-derived intestinal-type gastric cancer organoids—characterized by multilayered nuclei and multiple lumens—but was clearly distinct from the compact, poorly lumenized morphology observed in diffuse-type gastric cancer organoids (Figure 3*K*).

Finally, to visualize infection-induced structural disruption at high resolution, 2D organoids cultured under air–liquid interface conditions were exposed to *H pylori*

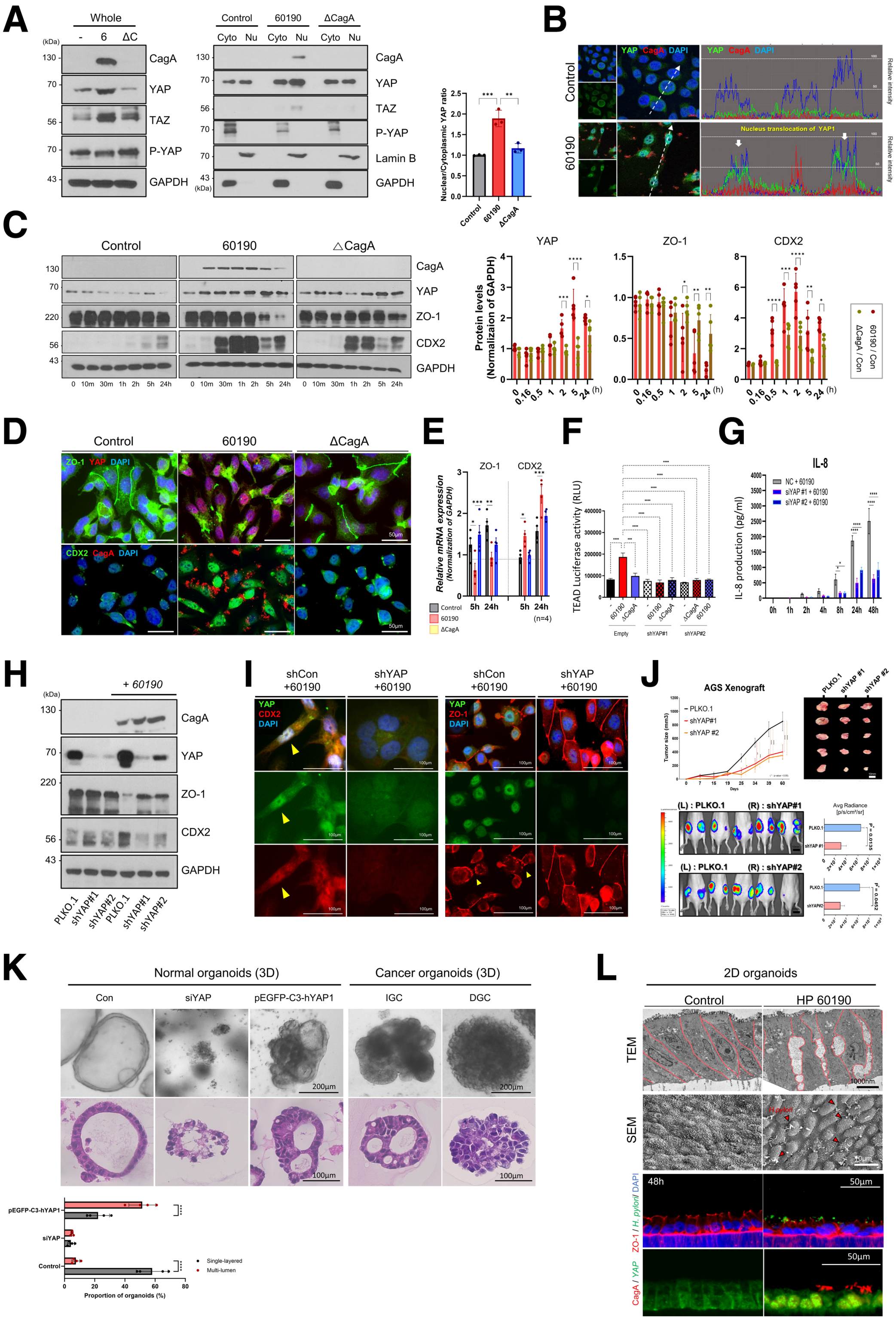

60,190 for 48 hours. This resulted in junctional rupture, expansion of intercellular spaces, and colocalization of YAP and CagA, as confirmed by transmission electron microscopy, scanning electron microscopy, and IF analysis (Figure 3*L*).

Collectively, these results demonstrate that *H pylori*–induced YAP nuclear translocation compromises epithelial tight-junction integrity and promotes intestinal-type metaplastic reprogramming.

## OTU Deubiquitinase, Ubiquitin Aldehyde Binding 2. –Mediated Yes-Associated Protein Activation via Deubiquitination and SUMOylation Drives Metaplastic Reprogramming

Given that YAP stability is regulated by post-translational ubiquitin modification, we next examined transcriptional changes in deubiquitination-related genes to identify potential upstream modulators of YAP under *H pylori* infection. Transcriptome analysis showed changes in the K63-linked deubiquitination gene set and highlighted OTUB2 (Figure 4*A*). By qRT-PCR, OTUB2 expression was significantly increased in cells infected with *H pylori* 60,190 ($CagA^{+}$), compared with both uninfected controls and ΔCagA-infected cells (Figure 4*B*), and IF demonstrated colocalization of OTUB2 with CagA (Figure 4*C*).

Treatment with recombinant OTUB2 (rOTUB2) alone elevated YAP and CDX2 levels while reducing ZO-1 levels (Figure 4*D* and *E*), whereas siOTUB2 pretreatment markedly attenuated the infection-induced increase in YAP levels. These findings suggest that OTUB2 is involved in the process of YAP stabilization induced by *H pylori* infection (Figure 4*F*).

Immunoprecipitation (IP) of YAP followed by immunoblotting for ubiquitin detected a high-molecular–weight smear of polyubiquitinated YAP that varied across conditions. The smear intensity was lower with *H pylori* 60,190 infection than with no infection or ΔCagA, indicating that YAP ubiquitination is selectively suppressed under *H pylori* 60,190 infection (Figure 4*G*). IP of OTUB2 and blotting with anti-SUMO2/3 identified higher-molecular–weight OTUB2 species consistent with SUMOylated OTUB2, which increased with *H pylori* 60,190 infection (Figure 4*H*). Upon treatment with TAK-981, an inhibitor of the SUMO activating enzyme, these SUMOylated OTUB2 bands markedly decreased, and the coprecipitation of OTUB2 with YAP observed with *H pylori* 60,190 was reduced (Figure 4*I*).

These findings indicate that OTUB2 undergoes regulated SUMO2/3 conjugation in response to *H pylori* infection and that this modification promotes the stability of the OTUB2–YAP complex during infection.

In single-cell datasets, OTUB2 transcripts were enriched in specific epithelial compartments, with prominent signals in IM-related cells (Figure 4*J*). In human tissue specimens, OTUB2 expression increased across pathologic stages, underscoring clinical relevance (Figure 4*K*). Together, these findings indicate that OTUB2 acts as a deubiquitinase hub that channels CagA signaling to YAP stabilization and nuclear translocation (Figure 4*L*).

## *Comparative* Helicobacter *Infection in a Chief Cell–Specific YAP Knockout Model*

SPEM is the metaplasia most consistently observed after *Helicobacter* infection in mice; thus, we prespecified SPEM as the primary histologic endpoint to test in vivo requirement of YAP signaling. As SPEM arises from the reprogramming of mature chief cells at the base of oxyntic corpus glands, our analyses focused on the corpus. We generated an inducible Mist1-creERT2$^{Cre/+}$;YAP$^{fl/fl}$ model in adults to selectively remove YAP in chief cells (Figure 5*A*). Mice were then orally gavaged for 8 weeks with *H pylori* 60,190 ($CagA^{+}$), ΔCagA, or *H felis* (Figure 5*B*), ensuring equivalent infection durations across groups. Because *H pylori* shows

**Figure 3.** (***See previous page***). **Tight-junction disruption and IM are associated with nuclear translocation of YAP.** (*A*) Representative WB of nuclear and cytoplasmic fractions showing YAP and phospho-YAP in AGS cells infected with *H pylori* 60,190 or ΔCagA for 5 hours (n = 3). (*B*) IF staining of YAP and CagA in AGS cells fixed 5 hours after *H pylori* 60,190 infection. Nuclear translocation of YAP was confirmed by fluorescence intensity analysis. Scale bar, 50 μm. (*C*) Representative WB of ZO-1 and CDX2 in AGS cells infected with *H pylori* 60,190 or ΔCagA (n = 5). (*D*) Representative IF images of ZO-1 (*green*) and CDX2 (*red*) in AGS cells incubated with *H pylori* 60,190 or ΔCagA for 5 hours; scale bar, 50 μm. (*E*) RT-PCR analysis of ZO-1 and CDX2 mRNA after infection with 60,190 or ΔCagA (n = 4). (*F*) TEAD luciferase reporter activity with *H pylori* infection and with YAP knockdown (n = 3). (*G*) IL-8 production in siYAP-treated AGS cells with or without infection (n = 5). (*H*) Representative WB of ZO-1 and CDX2 in YAP-silenced AGS cells (n = 3). (*I*) Representative IF images of ZO-1 (*red*), CDX2 (*red*), and YAP (*green*) in YAP-silenced AGS cells incubated with *H pylori* 60,190 for 5 hours. Scale bar, 100 μm. (*J*) In vivo xenograft tumor growth of AGS cells following YAP depletion. *Top left*: Longitudinal measurement of tumor volume in nude mice injected with pLKO.1 control, shYAP#1, or shYAP#2 AGS cells over a 60-day period. *Top right*: Representative images of excised xenograft tumors at the experimental endpoint. *Bottom left*: Representative bioluminescence images of tumor-bearing mice injected with pLKO.1 control or shYAP#1- or shYAP#2-expressing AGS cells. *Bottom right*: Quantification of bioluminescence signal shown as average radiance (photons/sec/$cm^2$/sr). n = 5 mice per group. Scale bar, 10 mm. (*K*) Representative images of normal and gastric cancer organoids after siYAP or YAP overexpression through electroporation; scale bars, 200 μm (*top*), 100 μm (*bottom*). The bar graph shows the proportion of single-layered and multilumen organoids (n = 5 independent organoid lines). (*L*) Transmission electron microscopy/scanning electron microscopy and IF analysis of ALI-cultured 2D organoids infected with 60,190 for 48 hours (ZO-1, *H pylori*, YAP, CagA); scale bars, 1000 nm (transmission electron microscopy), 10 μm (scanning electron microscopy), 50 μm (IF). Statistics: Data are presented as mean ± SEM. Multi-group/factorial data were analyzed by 1-way or 2-way ANOVA with Tukey's post hoc test; 2-group comparisons used 2-tailed unpaired Student's *t* tests. **P* < .05; ***P* < .01; ****P* < .001; *****P* < .0001. ALI, air–liquid interface. SEM, standard error of the mean.

limited colonization in mice, *H felis*, which can achieve stable infection and robust pathology, was included as a comparator species to enable more consistent evaluation of strain-dependent tissue responses. In corpus hematoxylin and eosin (H&E) sections, WT mice developed prominent inflammation and glandular distortion after infection with *H pylori* 60,190 and *H felis*, whereas these pathologic changes were markedly attenuated in knockout (KO) mice (Figure 5*C*). Notably, *H pylori* 60,190 ($CagA^{+}$) induced more pronounced lesions than its ΔCagA strain, underscoring the contribution of CagA to disease severity. Species-specific in situ hybridization (ISH) probes verified colonization in

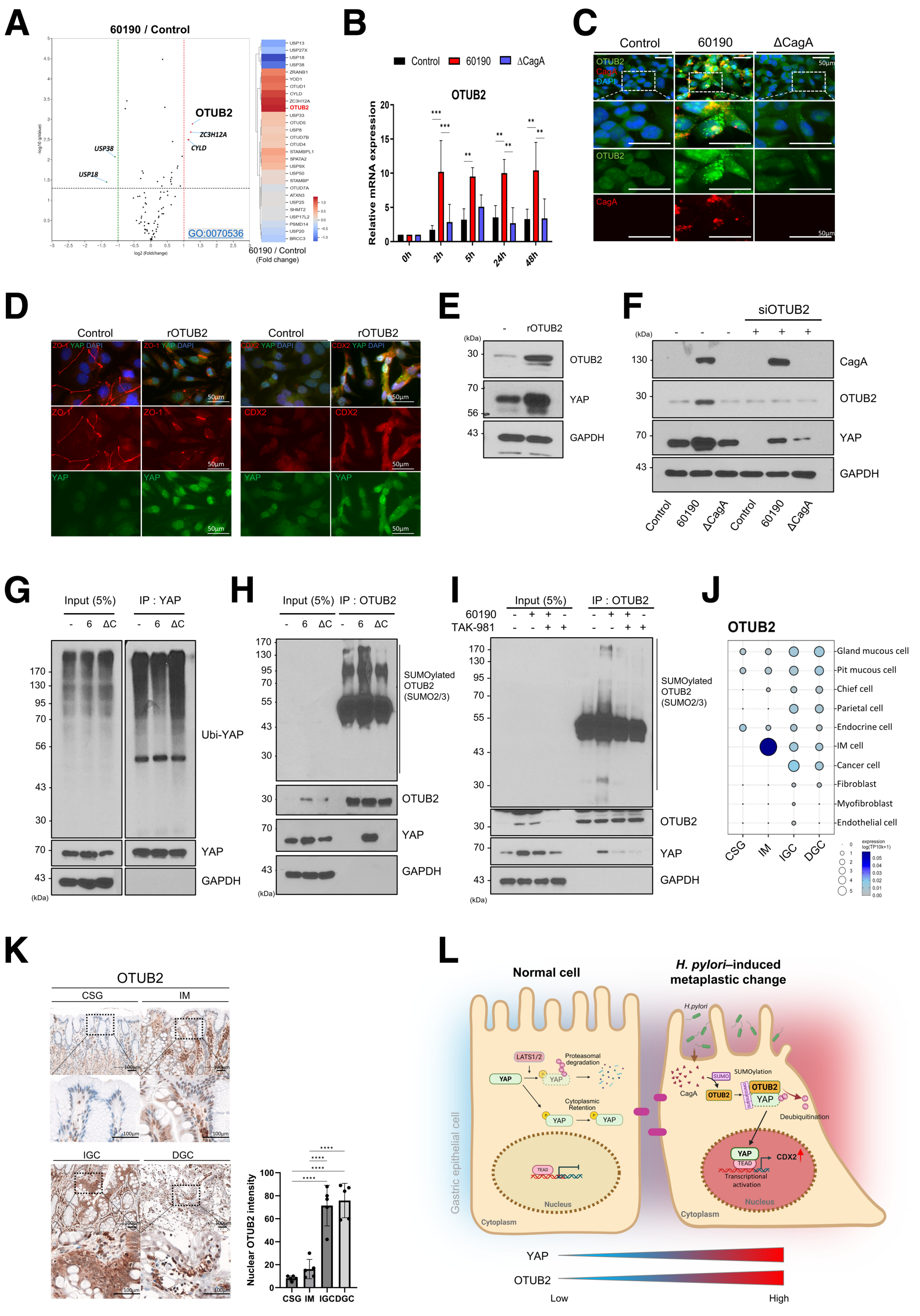

both genotypes; however, tissue damage and glandular alterations were strongly suppressed in KO animals (Figure 5*D*). Consistently, H&E-based inflammation scores were elevated in WT mice infected with either *H pylori* 60,190 or *H felis*, both showing significant increases relative to phosphate-buffered saline (PBS) or ΔCagA, whereas KO mice remained largely protected (Figure 5*E*). ISH quantification showed that WT mice infected with *H pylori* 60,190 exhibited higher gland-base colonization ratios than KO mice and that colonization was significantly greater following *H pylori* 60,190 infection, compared with the ΔCagA strain (Figure 5*F*). This analysis provides a framework for subsequent quantification of the SPEM pathology (parietal cell loss, expansion of the neck region, and a shift in the proliferative region) and suggests that strain virulence ($CagA^{+}$) and interspecies colonization capacity (*H felis*) may modulate the intensity of tissue responses at the site of chief cell reprogramming.

## *Yes-Associated Protein–Dependent Gastric Lineage Reprogramming Under Pathogenic* Helicobacter *Infection*

To further delineate how YAP influences epithelial hierarchy and lineage allocation in the gastric corpus, we analyzed a panel of markers representing proliferative, chief, mucous, and parietal cell compartments. These analyses aimed to determine whether YAP activation under infection shifts the balance from a differentiated to a regenerative or metaplastic state. In uninfected controls, Ki67-positive cells were confined to the neck and isthmus. Following infection, the proliferative region extended down toward the gland base, with significant expansion in *H pylori* 60,190 ($CagA^{+}$), but not in KO mice. Conversely, the ΔCagA strain produced no appreciable change, compared with PBS controls (Figure 6*A*). Markers of mature chief cells (MIST1 and pepsinogen [PGC]) decreased in WT mice upon infection with *H pylori* 60,190, but not with the ΔCagA strain, and these decreases were attenuated in KO mice (Figure 6*B* and *C*). The surface mucous marker MUC5AC expanded into deeper glands in WT mice following infection with *H pylori* 60,190 but remained at baseline with the ΔCagA strain and was suppressed in KO mice (Figure 6*D*). Consistently, $ATP4B^{+}$ parietal cells decreased in WT mice, whereas Griffonia simplicifolia II lectin (GSII) lectin binding expanded into the neck/base region, most prominently with *H pylori* 60,190 infection. Conversely, ΔCagA resembled PBS, and in KO mice, ATP4B was preserved with only limited GSII expansion (Figure 6*E*). Together, these coordinated changes indicate that YAP activation under pathogenic infection drives a broad remodeling of gastric lineage architecture—marked by loss of mature cell identity, expansion of proliferative and mucous compartments, and emergence of a reparative/metaplastic phenotype. Collectively, these lineage rearrangements were pronounced in WT mice but mitigated in chief cell–specific YAP KO mice despite identical infection stimuli, indicating that epithelial-intrinsic YAP activity amplifies tissue remodeling and that CagA is required for *H pylori*–driven changes. Notably, infection with *H felis*, a well-established murine-adapted *Helicobacter* species that robustly induces corpus pathology, consistently recapitulated these lineage alterations, thereby serving as a positive control confirming the YAP-dependent nature of pathogenic gastric remodeling in vivo.

## *Requirement of Yes-Associated Protein for Chief Cell–Derived Metaplasia (Spasmolytic Polypeptide–Expressing Metaplasia)*

As SPEM represents a prototypical injury-induced lineage reprogramming that arises from mature chief cells, we tested whether YAP activity within these cells is required for this transition. SPEM serves as a key intermediate state that bridges parietal cell loss and the emergence of intestinal-type lineages, reflecting an early adaptive response that can progress toward preneoplastic remodeling. Therefore, a chief cell–specific YAP KO model provides a direct means to determine whether YAP is indispensable for initiating this regenerative-to-metaplastic switch under chronic *Helicobacter* infection. We evaluated GSII–gastric intrinsic factor (GIF) double positivity (a hallmark of SPEM), CD44 variant 9 (CD44v9; damage/repair–metaplasia marker), and TFF2 (a trefoil factor expressed by mucous neck and SPEM cells). In WT mice, infection with *H pylori* 60,190 ($CagA^{+}$) and with *H felis*

**Figure 4.** (***See previous page***). **OTUB2-mediated YAP stabilization through deubiquitination and SUMOylation.** (*A*) Scatter and heatmap of genes in K63-linked deubiquitination (GO:0070536) from RNA-seq in AGS cells infected with *H pylori* 60,190; OTUB2 highlighted. (*B*) qRT-PCR for OTUB2 in AGS cells infected with 60,190 or ΔCagA (GAPDH-normalized; n = 4). (*C*) Representative IF images for OTUB2 (*green*) and CagA (*red*) after 5-hour infection in AGS cells; scale bar, 50 μm. (*D*) Representative IF images for YAP (*green*), CDX2 (*red*), and ZO-1 (*red*) in AGS cells after recombinant OTUB2 (16 μg/mL) treatment; scale bar, 50 μm. (*E*) Representative WB for YAP, CDX2, and ZO-1 in AGS cells after rOTUB2 (16 μg/mL) (n = 3). (*F*) Representative WB for OTUB2 and YAP in AGS cells after siOTUB2 pretreatment followed by *H pylori* infection (n = 3). (*G*) Representative IP–WB detecting YAP ubiquitination in AGS cells (n = 3). (*H*) Representative WB analysis of SUMO2/3 signals in total cell lysates and OTUB2 immunoprecipitates from AGS cells to assess OTUB2 SUMOylation under *H pylori* infection (n = 3). (*I*) Representative WB showing the effect of the SUMO-activating enzyme inhibitor TAK-981 on OTUB2 SUMOylation and its association with YAP in AGS cells (n = 3). (*J*) Dot plot of OTUB2 expression across gastric epithelial cell types and pathologic stages. (*K*) OTUB2 immunohistochemistry in human CSG, IM, IGC, and DGC samples; scale bar, 100 μm. (*L*) Schematic of CagA-driven activation of YAP signaling promoting metaplastic changes in the gastric epithelium. Statistics: Data are presented as mean ± SEM. One-way or 2-way ANOVA with Tukey's post hoc test. **P* < .05; ***P* < .01; ****P* < .001; *****P* < .0001. CSG, chronic superficial gastritis; DGC, diffuse-type gastric cancer; IGC, intestinal-type gastric cancer; SEM, standard error of the mean.

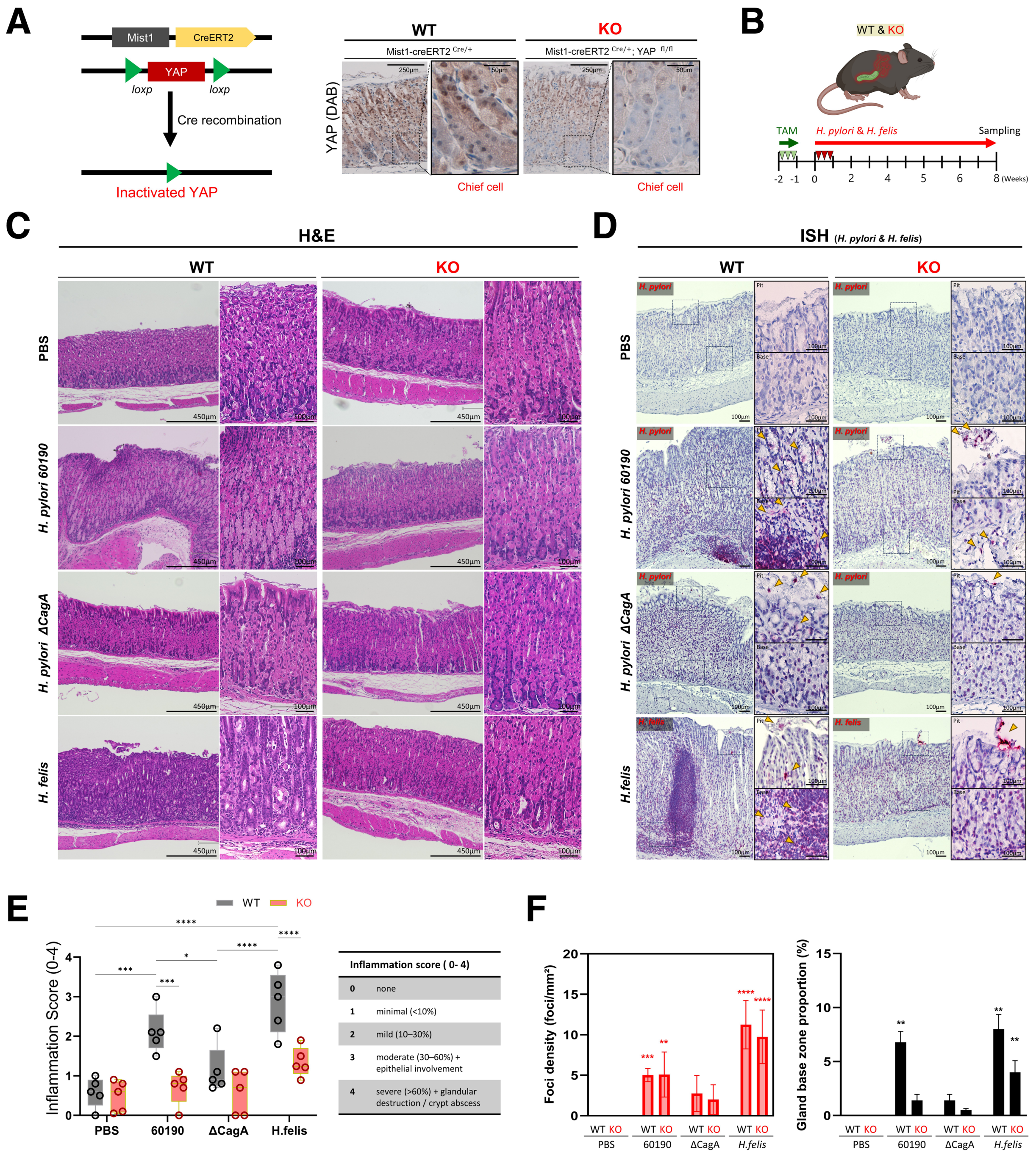


**Figure 5. Tissue-level changes with chief cell–specific YAP manipulation and comparative *Helicobacter* infection.** (*A*) Schematic of chief cell–specific conditional deletion using Mist1-creERT2$^{Cre/+}$;YAP$^{fl/fl}$, with YAP loss by IHC at the gland base after tamoxifen (*inset*). (*B*) Eight-week infection design in WT (Mist1-creERT2$^{Cre/+}$) and chief cell–specific YAP KO (Mist1-creERT2$^{Cre/+}$;YAP$^{fl/fl}$): PBS, *H pylori* 60,190 (CagA$^{+}$), ΔCagA, or *H felis*. (*C*) Representative H&E of oxyntic corpus at 8 weeks; scale bars, 450 $\mu$m and 100 $\mu$m. (*D*) Representative ISH with species-specific probes for *H pylori* and *H felis* (*red*) in the gastric corpus, counterstained with hematoxylin; scale bar, 100 $\mu$m. (*E*) Inflammation scores (0–4; 0 none, 1 minimal, 2 mild, 3 moderate, 4 severe) in the corpus based on H&E (n = 5). (*F*) ISH-based quantification of foci density (foci/mm$^2$) and gastric gland-base colonization (%) for *H pylori* and *H felis* (n = 4). The *P* value indicates comparison with the control group. Statistics: Data are presented as mean ± SEM. Two-way ANOVA with Tukey's post hoc test. **P* < .05; ***P* < .01; ****P* < .001; *****P* < .0001. SEM, standard error of the mean.

significantly increased GSII$^{+}$/GIF$^{+}$ cells as a fraction of total GIF$^{+}$ chief cells (Figure 7*A*). Coexpression of YAP and CD44v9 also increased significantly, supporting the activation of chief cell–derived metaplastic change (Figure 7*B*). Similarly, TFF2 levels significantly increased under the same conditions, reinforcing the histologic

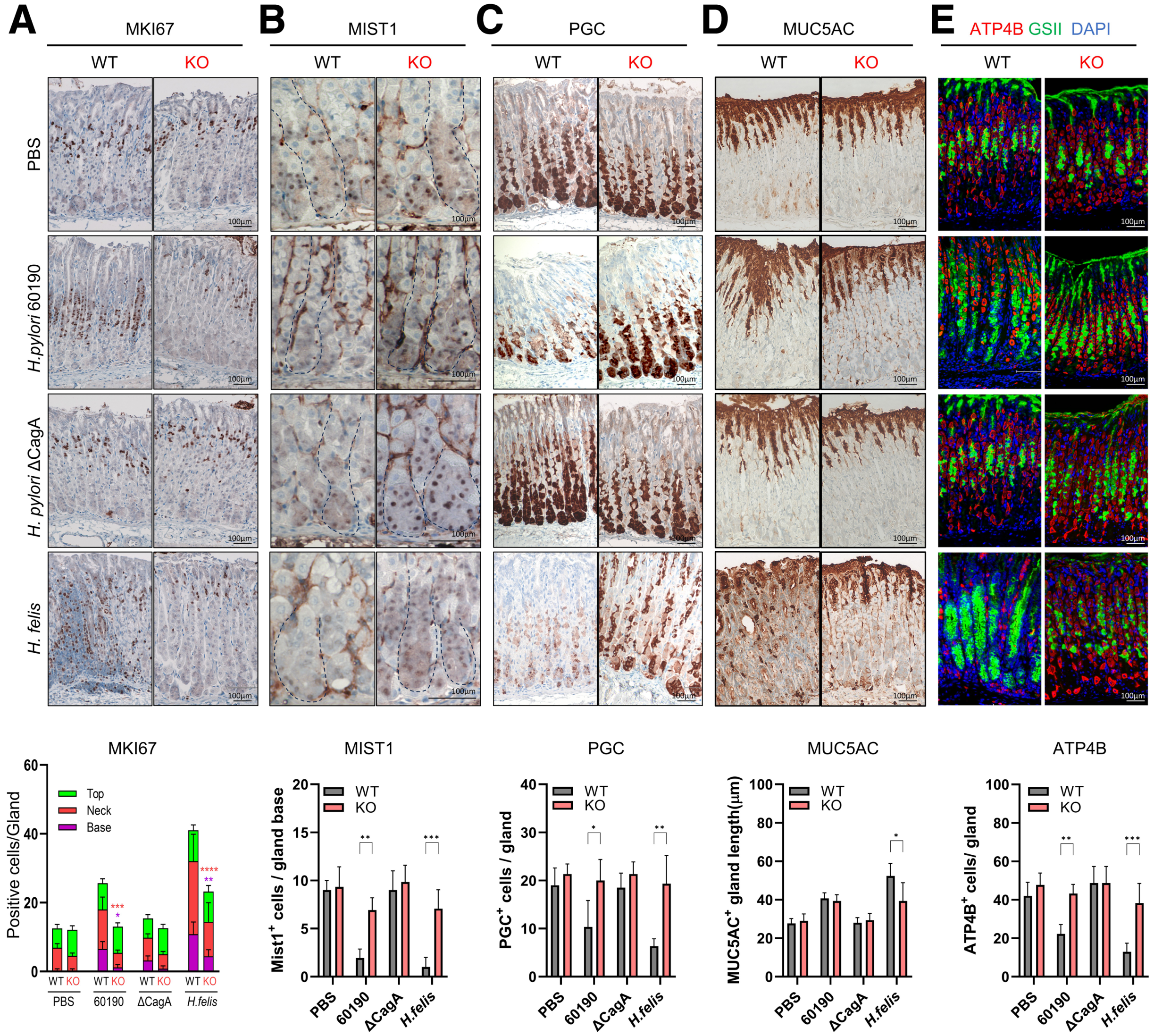


**Figure 6. Gastric lineage marker rearrangement in the corpus of WT and chief cell–specific YAP KO mice infected with *H pylori* 60,190 (CagA$^+$), ΔCagA, or *H felis*.** (*A–D*) Representative DAB IHC staining in the gastric corpus for (*A*) Ki-67 (proliferation), (*B*) MIST1 (chief cells), (*C*) PGC (chief cells), (*D*) MUC5AC (surface mucous cells); scale bar, 100 $\mu$m. (*E*) IF for ATP4B (parietal cells, *red*) and GSII lectin (neck mucous cells, *green*); scale bar, 100 $\mu$m. Quantifications are shown below each panel. Statistics: Data are presented as mean ± SEM. Two-way ANOVA with Tukey's post hoc test for genotype × infection factorial comparisons (n = 3). **P* < .05; ***P* < .01; ****P* < .001; *****P* < .0001. SEM, standard error of the mean.

consistency of SPEM (Figure 7*C*). Conversely, infection with the ΔCagA strain produced results comparable to PBS controls, with no significant induction of SPEM markers. In the chief cell–specific YAP knockout, GSII–GIF double positivity, CD44v9, and TFF2 levels remained near baseline, and infection-induced increases were significantly blunted (Figure 6*A–C*). This attenuation was accompanied by the preservation of glandular morphology and a restricted expansion of the proliferative zone, indicating that YAP loss constrains both structural and transcriptional aspects of metaplastic remodeling. This indicates a genotype-by-infection interaction and demonstrates that YAP is required to initiate chief cell reprogramming. Collectively, these findings reaffirm that the SPEM program triggered by *H pylori* infection is YAP-dependent.

## *Pharmacologic Modulation of OTU Deubiquitinase, Ubiquitin Aldehyde Binding 2 Reveals the Plasticity of Yes-Associated Protein–Driven Metaplastic Signaling*

To determine whether YAP signaling, regulated by OTUB2, is pharmacologically tunable, we tested gain- and loss-of-function interventions in vivo. Repeated intraperitoneal dosing of rOTUB2 (2 $\mu$g/100 $\mu$L in PBS, every other day for 7 doses) enhanced YAP and CD44v9 expression in the oxyntic corpus, accompanied by a shift of ATP4B–GSII markers toward a metaplastic pattern (Figure 8*A–C*). This treatment also increased the number of GSII$^+$ cells, indicating that enforced OTUB2 activity amplifies YAP-driven reprogramming and proliferation.

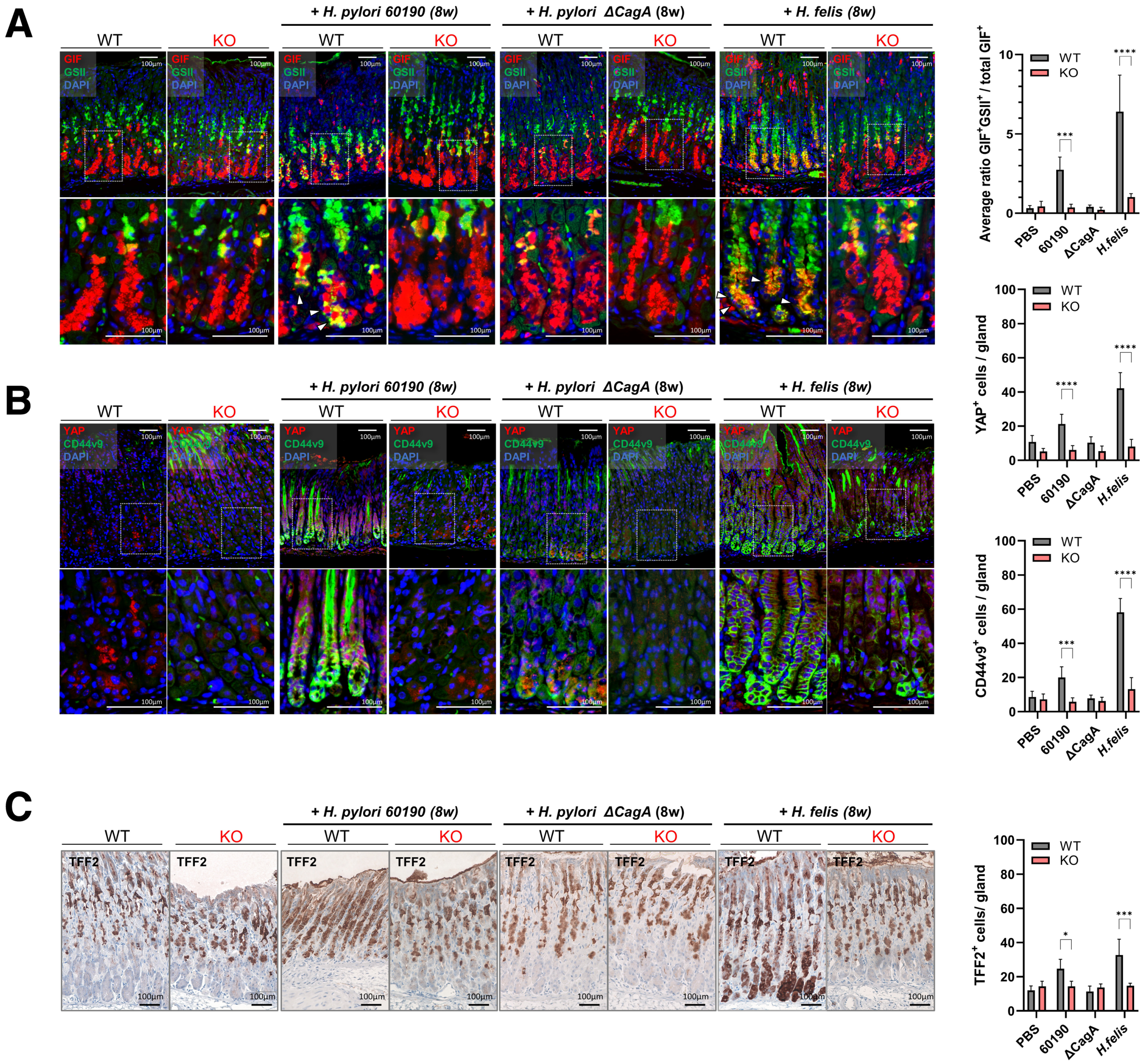


**Figure 7. Chief cell–specific YAP KO suppresses SPEM in the oxyntic corpus during *H pylori* and *H felis* infection.** SPEM was assessed based on GSII–GIF double positivity and the expression of established metaplasia-associated markers, including CD44v9 and TFF2. (*A*) Representative IF for GSII and GIF in WT and chief cell–specific YAP KO mice infected with *H pylori* 60,190 (CagA+), ΔCagA, or *H felis*. GSII+/GIF+ double-positive cells per gland were quantified (n = 5). Scale bar, 100 $\mu$m. (*B*) Representative IF for YAP and CD44v9 in the same corpus samples; YAP+ and CD44v9+ cells per sample were quantified (n = 5). Scale bar, 100 $\mu$m. (*C*) Representative TFF2 DAB IHC under the same conditions (n = 3). Scale bar, 100 $\mu$m. Statistics: Data are presented as mean ± SEM. Two-way ANOVA with Tukey's post hoc test for genotype × infection comparisons. **P* < .05; ***P* < .01; ****P* < .001; *****P* < .0001. SEM, standard error of the mean.

In contrast, in chief cell–specific YAP-deficient mice (Mist1-creERT2$^{Cre/+}$;YAP$^{fl/fl}$), rOTUB2 administration failed to induce the increase in YAP and CD44v9 expression, parietal cell (ATP4B) loss, or GSII expansion observed in WT mice (Figure 8*B*), indicating that OTUB2-induced metaplastic changes occur in a YAP-dependent manner in vivo.

Conversely, pretreatment with the selective OTUB2 inhibitor LN5P45 (2.5 mg/100 $\mu$L intraperitoneally) prior to *H pylori* 60,190 infection markedly diminished YAP, OTUB2, and CD44v9 signals, while maintaining relatively preserved ATP4B–GSII indices (Figure 8*D*–*F*). The inhibitory effect of LN5P45 also limited the expansion of the proliferative zone and preserved glandular polarity, underscoring the functional reversibility of this signaling axis. These reciprocal effects demonstrate bidirectional control of YAP activity and highlight OTUB2 as an upstream modulator of YAP stabilization in vivo.

Together with genetic results of the genetic analysis, these data indicate that the pharmacologic perturbation of OTUB2 can dynamically reshape the epithelial response to *H pylori* infection. Therefore, OTUB2 activation promotes the YAP-dependent epithelial metaplastic program, whereas OTUB2 inhibition or YAP loss suppresses it. Our

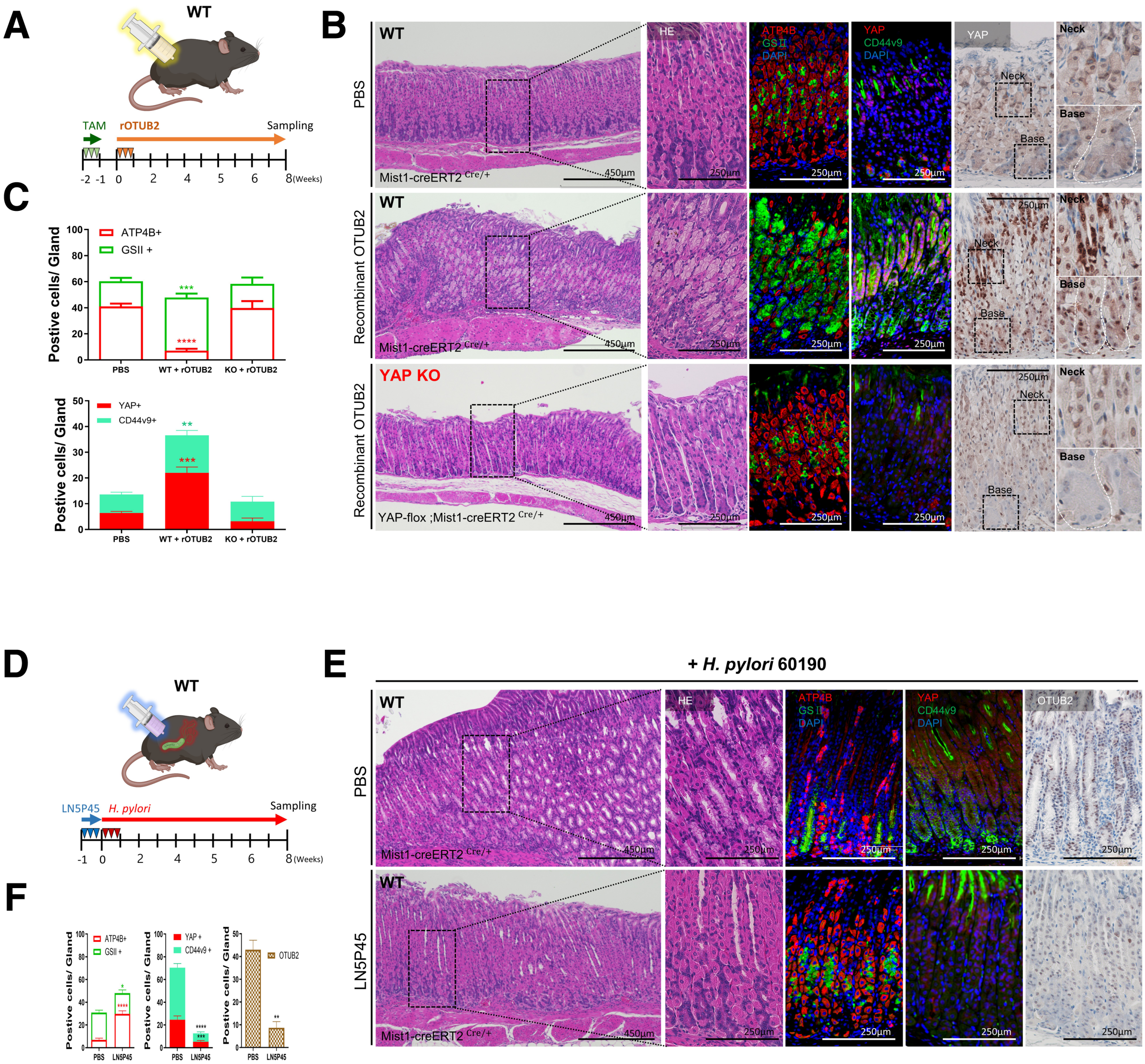


**Figure 8. Pharmacologic modulation of OTUB2 regulates YAP-dependent SPEM.** (*A*) rOTUB2 dosing scheme: 7 doses of rOTUB2 (2 $\mu$g/100 $\mu$L in PBS) were intraperitoneally administered every other day. (*B*) Representative corpus IF staining in WT mice and in chief cell–specific YAP KO mice following rOTUB2 treatment: CD44v9 (*green*), ATP4B (*red*), GSII (*green*), and YAP (*red*, DAB); scale bar, 450 $\mu$m and 250 $\mu$m. (*C*) Quantification of positive cells for the markers in (*B*) (n = 5). (*D*) Inhibitor scheme: LN5P45 (2.5 mg/100 $\mu$L, intraperitoneal) pretreatment followed by *H pylori* infection. (*E*) Representative corpus IF stains after LN5P45 + *H pylori* 60,190: CD44v9 (*green*), ATP4B (*red*), GSII (*green*), YAP (*red*), and OTUB2 (DAB); scale bar, 450 $\mu$m and 250 $\mu$m. (*F*) Quantification of positive cells in (*E*) (n = 5). Statistics: Data are presented as mean ± SEM. Two-way ANOVA with Tukey's post hoc test for factorial comparisons (eg, treatment × infection); 2-tailed unpaired Student's *t* tests for 2-group comparisons. *$P < .05$; **$P < .01$; ***$P < .001$; ****$P < .0001$. SEM, standard error of the mean.

findings confirm that epithelial-intrinsic and tissue-level reprogramming converge on YAP signaling, with OTUB2 acting as a regulatory amplifier.

## Discussion

In this study, we functionally validated a CagA–OTUB2–YAP signaling axis as the molecular pathway through which *H pylori* infection drives epithelial junctional disruption and metaplastic reprogramming in the gastric epithelium.[12,20] By integrating human epithelial models, single-cell transcriptomic analyses, and in vivo genetic approaches, we demonstrated that CagA-dependent signals promote YAP stabilization through OTUB2 and that epithelial-intrinsic YAP activity plays a functionally important role in *Helicobacter*-induced metaplastic responses. These findings position YAP as a central regulator of infection-associated epithelial plasticity and provide a mechanistic framework linking bacterial virulence to early gastric disease progression.[18,23]

Although YAP activation has been repeatedly observed in gastric tissues from patients with chronic *H pylori* infection, prior studies have largely described this association at a correlative level.[19,20] Herein, we showed that infection with CagA-positive *H pylori* robustly induces YAP stabilization and nuclear accumulation, whereas these effects are markedly attenuated in ΔCagA infection.[18] Moreover, the depletion of OTUB2 substantially reduced CagA-induced YAP stabilization, supporting a role for OTUB2 as a functional regulator that transduces CagA signaling to YAP activation.

OTUB2 suppresses YAP degradation by removing K48-linked ubiquitin chains.[24,25] Our findings extend this model by demonstrating that OTUB2 acts as a functional regulator of YAP stability in the context of CagA-positive *H pylori* infection. Under CagA-positive infection conditions, reduced YAP ubiquitination coincided with increased OTUB2 SUMOylation, which was sensitive to inhibition by the SUMO E1 inhibitor TAK-981. Although the precise molecular consequences of OTUB2 SUMOylation were not directly unraveled in this study, SUMOylation modulates protein stability, enzymatic activity, and protein–protein interactions, which highlight the plausibility of this modification enhancing OTUB2 function under infection-induced stress.[40,41]

At the transcriptional level, YAP activation in human gastric epithelial cell lines was associated with a clear epithelial-intrinsic "IM-like" transcriptional shift, characterized by the loss of ZO-1, induction of CDX2, and activation of TEAD-dependent transcription.[19,32,42] YAP activity peaked approximately 5 hours after *H pylori* infection and subsequently declined, while remaining elevated relative to baseline, indicating that YAP is most strongly engaged during the early phase of infection. These findings support a model in which CagA disrupts junctional and inflammatory signaling circuits, mobilizes YAP to the nucleus, and transiently opens an intestinal-type transcriptional program.[4,8,9,42]

To connect these epithelial-intrinsic transcriptional changes to tissue-level pathology, we focused on the mouse oxyntic corpus and analyzed SPEM as a principal indicator of chief-cell reprogramming.[35–37,43] Infection with CagA-positive *H pylori* was associated with parietal cell loss, expansion of the neck region, downward displacement of the proliferative zone, and induction of established SPEM markers, including GSII–GIF, CD44v9, and TFF2. In contrast, these metaplastic responses were markedly reduced in chief cell-specific YAP KO mice, demonstrating that epithelial YAP activity is functionally required for SPEM induction.[22]

Although GKN3 has been proposed as a marker of SPEM, particularly in murine studies, its interpretation in human datasets requires caution, as the human ortholog is annotated as a pseudogene (GKN3P) with uncertain functional conservation.[44] In addition, GKN3/GKN3P expression is variably detected in human gastric epithelial populations and is often sparse in single-cell transcriptomic datasets.[38] Consistent with this, GKN3/GKN3P was not robustly detected in our dataset. Therefore, we defined epithelial cell identities based on combinatorial marker expression and overall transcriptional context rather than relying on a single marker.[38,39]

Metaplastic changes were consistently more severe following infection with *H pylori* 60,190 (CagA$^+$) than with the ΔCagA strain, supporting a role of CagA as a disease-modifying factor that amplifies epithelial reprogramming in the context of *H pylori*-induced injury.[45] In contrast, infection with *H felis*—a rodent-adapted gastric *Helicobacter* species that does not encode CagA—markedly induced more pronounced metaplastic changes, demonstrating that lesion severity can be strongly influenced by species-specific pathogenic properties. Accordingly, in this study, *H felis* was not used to model CagA-dependent mechanisms, but instead served as a rodent-adapted reference (positive control) model that reliably elicits robust inflammatory and metaplastic responses in the murine stomach.[34,46,47] In chief cell–specific YAP KO mice, the observation that SPEM was reduced but not completely abolished can be reasonably explained by incomplete tamoxifen-induced recombination and cellular heterogeneity within the gastric epithelium.[34,35,48] Although the cellular origin of SPEM remains debatable, multiple lineage-tracing studies have consistently demonstrated that mature chief cells represent a major cellular source of SPEM.[23,36,37,43] In this context, our genetic data strongly support a central role for chief cell–intrinsic YAP signaling in *Helicobacter*-induced metaplastic reprogramming.[21,22]

These findings are also consistent with the concept of paligenosis, as proposed by the Mills laboratory, wherein differentiated chief cells respond to injury by undergoing dedifferentiation and transcriptional reprogramming en route to a metaplastic state.[49–51] Our data suggest that activation of the CagA–OTUB2–YAP axis represents a molecular mechanism through which microbial infection engages this injury-associated epithelial plasticity program.[23,24,49]

Beyond its role in cell fate reprogramming, our data further suggest that YAP activation may also amplify inflammatory and tissue remodeling responses. Although inflammation and oxyntic atrophy are well-established upstream drivers of metaplastic reprogramming,[23] our data indicate that epithelial-intrinsic YAP activity contributes to sustaining these processes. In vitro, YAP activation was associated with increased expression of inflammatory mediators, including IL-8, and disruption of epithelial barrier integrity, whereas YAP depletion attenuated these responses. These findings support a model in which YAP reinforces epithelial injury signals, thereby promoting sustained inflammatory and metaplastic progression. Accordingly, loss of YAP in chief cells may attenuate epithelial-derived inflammatory signaling, leading to reduced tissue-level inflammation and oxyntic atrophy. From a translational perspective, YAP forms a nexus at which injury responses, regeneration, and metaplasia converge during chronic *H pylori* infection.[18,21,22] By identifying OTUB2 as an upstream regulator of YAP stability, our study highlights potential avenues for therapeutic intervention, including OTUB2 inhibition or YAP pathway

blockade.[14,26,27] In addition, the coexpression of OTUB2, YAP, and SPEM markers may inform pathology- and transcriptome-based strategies for early risk-stratification.[22,38,39]

In summary, this study establishes YAP as a central regulator of *H pylori*-induced epithelial metaplastic reprogramming and delineates a CagA–OTUB2–YAP signaling axis that links epithelial junctional disruption and transcriptional remodeling in cells and organoids to chief cell–derived SPEM in vivo.[19,21,22] This pathway represents a common denominator of infection-associated epithelial plasticity and a therapeutically actionable target, providing a molecular foundation for early intervention and prevention strategies in gastric cancer.

# Materials and Methods

## Human Gastric Tissues

Human gastric tissue specimens, including chronic superficial gastritis, IM, and gastric cancer, were obtained from patients at Severance Hospital, Yonsei University College of Medicine. All patients provided informed consent, and the protocols were approved by the Institutional Review Board (IRB; Nos. 4-2011-0149, 4-2013-0880, and 4-2018-0676).

## Cell Culture and Helicobacter Infection

Human gastric cancer cell lines AGS (CRL-1739, ATCC, RRID:CVCL_0139), AGS-LUC2 (CRL-1739-LUC2, ATCC, RRID:CVCL_A4BR), MKN74 (80,104, KCLB, RRID:CVCL_2791), N87 (CRL-5822, ATCC, CVCL_1603), KATO III (HTB-103, ATCC, RRID:CVCL_0371), the colorectal cancer cell line Caco-2 (HTB-37, ATCC, RRID:CVCL_0025), and HEK293T (CRL-11268, ATCC RRID:CVCL_1926) were cultured in RPMI-1640 (HyClone, SH30027.01) or Dulbecco's Modified Eagle Medium (HyClone, SH30243.01) supplemented with 10% fetal bovine serum (Thermo Fisher Scientific) and 1 % penicillin–streptomycin (Thermo Fisher Scientific) at 37 °C in a humidified 5% $CO_2$ incubator.

*H pylori* strains—60190 ($CagA^+$, ATCC 49503), ΔCagA mutant (CagA-deletion strain, kindly provided by Dr Masanori Hatakeyama, University of Tokyo), 11,637 ($CagA^+$, ATCC 43504), and 8822 ($CagA^-$, ATCC 51932)—and *H felis* (ATCC 49179) were cultured on Brucella agar plates supplemented with 10% sheep blood under microaerophilic conditions at 37 °C using the Campy Container system (BBL). For infection experiments, bacterial cells were harvested, suspended in Dulbecco's PBS (pH 7.4), and adjusted to an optical density of $OD_{600}$ corresponding to a multiplicity of infection (MOI) of 100. Control cells were treated with PBS under the same conditions.

## Gastric Organoid Culture

Patient-derived gastric organoids were established from gastric tissue biopsies obtained with IRB approval, following previously described protocols.[5,29,30] Organoids were embedded in Matrigel (Corning #356231) and cultured in Advanced Dulbecco's Modified Eagle Medium/F12 (Gibco, Thermo Fisher Scientific) supplemented with penicillin–streptomycin (Gibco #15140-122), HEPES (10 mM; Invitrogen), GlutaMAX (Invitrogen #35050-061), B27 supplement (Invitrogen #17504-044), and N-acetylcysteine (1 mM; Sigma-Aldrich #A7250). Growth factors were added as follows: epidermal growth factor (50 ng/mL; PeproTech #AF-100-15), Noggin (PeproTech #120-10C), R-spondin1 conditioned medium (10%), Wnt-conditioned medium (50%), fibroblast growth factor 10 (200 ng/mL; PeproTech #100-26), gastrin (1 nM; Tocris #3006), and transforming growth factor $\beta$ inhibitor A83-01 (2 $\mu$M; Sigma-Aldrich #SML0788). Optional supplements included nicotinamide (10 mM; Sigma-Aldrich #N0636) and ROCK inhibitor Y-27632 (10 $\mu$M; Sigma-Aldrich #Y0503). For 2D culture, organoid-derived single cells were seeded on collagen type I–coated Transwell inserts (15 $\mu g/cm^2$; Corning #3470) and cultured under air–liquid interface. *H pylori* infection of 2D organoids was performed on day 7 of culture at an MOI of 100 to 200 using *H pylori* 60,190 ($CagA^+$) or ΔCagA suspended in PBS.

## Mouse Models

Eight- to 10-week-old C57BL/6J mice, kindly provided by Professor. Ki Taek Nam (Yonsei University), were used. Mist1-creERT2$^{Cre/+}$ mice were generated using standard embryonic stem cell–targeting techniques, in which the entire coding region of the Mist1 gene was replaced with CreERT2. YAP$^{fl/fl}$ mice were derived from YAP/TAZ$^{fl/fl}$ mice originally obtained from The Jackson Laboratory (JAX #030532). To generate a chief cell–specific Yap KO, Mist1-creERT2$^{Cre/+}$; YAP$^{fl/fl}$ mice were subcutaneously treated with 3 doses of 5 mg tamoxifen. The tamoxifen dosing regimen was selected based on prior studies to achieve efficient recombination in Mist1$^+$ chief cells. Mice were infected with *H pylori* 60,190 ($CagA^+$ or ΔCagA) or *H felis* through oral gavage for 8 weeks. All the animal experiments were performed in accordance with institutional guidelines and approved by the Institutional Animal Care and Use Committee (IACUC No. 2022-0314, Yonsei University).

## Modulation of OTU Deubiquitinase, Ubiquitin Aldehyde Binding 2 Activity In Vivo

To evaluate the functional role of OTUB2 in gastric metaplastic responses, rOTUB2 (2 $\mu$g/100 $\mu$L; Sino Biological, #13177-H07E) or the OTUB2 inhibitor LN5P45 (2.5 mg/100 $\mu$L; MedChemExpress, #HY-149482) was intraperitoneally administered to C57BL/6J mice every other day for 7 days. WT mice received rOTUB2 to assess its ability to promote SPEM, whereas *H pylori*–infected mice were pretreated with LN5P45 to examine the inhibition of OTUB2-mediated metaplastic signaling. Stomach tissues were collected 8 weeks after the final injection. Control groups received equal volumes of PBS.

## Lentiviral Transduction and Generation of Stable Cell Lines

Premade lentiviral short-hairpin RNA (shRNA) constructs and a negative control consisting of a construct identical to the vector system (pLKO.1) were purchased

from Addgene (shYAP#1, 42,540; shYAP#2, 42,541; shControl, 8453). Lentiviral particles were produced in vitro using a 3rd Generation Packaging System mix (LV053, Abcam) and lentipectin (G074, Applied Biological Materials). According to the manufacturer's protocol, cell supernatants were harvested 48 hours after transfection in HEK293T cells and used to infect cells or stored at −80 °C. Puromycin (2 μg/mL) was used 48 hours after lentiviral infection to select AGS and MKN74 cells that stably expressed shRNA constructs. Western blotting (WB) was used to identify and validate the positive clones.

### Xenograft Mouse Model and Bioluminescence Imaging

AGS cells ($5 \times 10^6$) stably expressing shRNA targeting YAP (shYAP#1 or shYAP#2) or control shRNA (pLKO.1) were subcutaneously injected into the hind limbs of BALB/c nude mice. Mice were maintained in a pathogen-free animal facility at Yonsei University College of Medicine. All animal procedures were approved by the Institutional Animal Care and Use Committee (IACUC no. 2023-0047).

Tumor growth was monitored every 3 to 5 days using caliper measurements, and tumor volume was calculated using the formula $(\text{length} \times \text{width}^2)/2$. At day 60 after injection, the mice were intraperitoneally injected with D-luciferin (150 mg/kg body weight; dissolved in sterile PBS at 15 mg/mL) for bioluminescence imaging. Luminescent signals were acquired 10 to 15 minutes after injection using an IVIS Spectrum imaging system (PerkinElmer).

At the experimental endpoint, tumors were excised for gross imaging. Tumor tissues were either snap-frozen in liquid nitrogen or fixed in 10% formaldehyde for subsequent immunohistochemistry (IHC) and H&E staining.

### Yes-Associated Protein–TEA Domain Transcription Factor Luciferase Assay

The cells were transduced at an MOI of 5 using a commercial lentiviral construct incorporating a luciferase reporter for TEAD activity (TEAD Luciferase Reporter Lentivirus, 79,833, BPS Bioscience). A commercial kit (Steady-Glo Luciferase Assay System, E2510, Promega) was used to assess luciferase activity, which was normalized to the cell number.

### Enzyme-Linked Immunosorbent Assay

The concentration of IL-8 was measured using direct sandwich ELISA. Each sample was analyzed in quintuplicates. Human CXCL8/IL-8 was quantified using the DuoSET ELISA (DY208-05, R&D Systems) and DuoSET Ancillary Reagent (DY008, R&D Systems) kits. The absorbance was measured at 450 nm using a VersaMax microplate reader. The minimum detection sensitivity of ELISA was 20 pg/mL.

### Small interfering RNA transfection

YAP targeting and control small interfering RNAs (siRNAs) were synthesized by Bioneer (Daejeon, Korea). The sequences used were as follows:

Yes-associated protein small interfering RNA #1

- Sense: 5′-CAG AAG AUC AAA GCU ACU U-3′
- Antisense: 5′-AAG UAG CUU UGA UCU G-3′

Yes-associated protein small interfering RNA #2

- Sense: 5′-AGA ACC GUU UCC CAG ACU A-3′
- Antisense: 5′- UCU GGG AAA CGG UUC U-3

For OTUB2 knockdown, a predesigned siRNA (sc-76016) was purchased from Santa Cruz Biotechnology. Transfection was performed using Lipofectamine RNAiMAX (13,778-150, Invitrogen) according to the manufacturer's instructions, with 20 nM siRNA applied to AGS cells.

### Plasmid Transfection

Following the manufacturer's instructions, Lipofectamine 2000 (11,668-019, Invitrogen) was used to transfect AGS cells with each construct. pEGFP-C3-hYAP1 (RRID:Addgene_17843) was purchased from Addgene. Full-length CagA (AF202923) and EPIYA phosphorylation-resistant mutants were provided by Professor M. Hatakeyama of the University of Tokyo. The medium was replaced with fresh medium 6 hours after transfection. Cells were used for all assays 24 hours after transfection.

### Electroporation

The YAP-EGFP vector and YAP siRNA were delivered to normal gastric organoids cultured for more than 7 days through electroporation using NEPA21 (Nepagene), according to the manufacturer's instructions.

### Cell Fractionation

AGS cells were infected with *H pylori* 60,190 (CagA-positive) or *H pylori* ΔCagA (CagA-negative). Following the manufacturer's instructions, nuclear-cytoplasmic fractionation was performed 5 hours after infection using the NE-PER Nuclear and Cytoplasmic Extraction Reagent Kit (78,833, Thermo Fisher Scientific).

### Immunoprecipitation Assay

AGS cells were transfected with His-tagged ubiquitin (Addgene #99540) for 24 hours prior to infection. Following *H pylori* infection, YAP or OTUB2 was immunoprecipitated using Protein A/G PLUS agarose beads. WB was performed using anti-Ub and anti-SUMO2/3 antibodies to detect polyubiquitinated YAP and SUMOylated OTUB2, respectively.

### Western Blotting

Cells were collected and resuspended in radioimmunoprecipitation assay buffer containing 25 mmol/L Tris-Cl (pH 7.4), 0.15 mol/L NaCl, 1% NP40, 0.1% sodium dodecyl sulfate (SDS), 1% sodium deoxycholate, and 1 mmol/L EDTA, along with a cocktail of protease and phosphatase inhibitors (P3300; GenDEPOT). The protein

concentrations in the lysates were measured, and an SDS sample buffer consisting of 62.5 mM Tris-Cl (pH 6.8), 2% SDS, 10% glycerol, β-mercaptoethanol, and 0.002% bromophenol blue was added. The samples were then heated to 95 °C for 5 minutes. Proteins were separated by SDS–polyacrylamide gel electrophoresis using 7% or 10% gels and subsequently transferred to Immobilon-FL polyvinylidene fluoride membranes (IPFL00010, Merck Millipore). Primary antibodies were incubated overnight at 4 °C with gentle agitation, and the signal was developed using the SuperSignal West Pico PLUS Chemiluminescent Substrate (34,580, Thermo Fisher Scientific) in the dark.

## Immunostaining and In Situ Hybridization

Formalin-fixed paraffin-embedded slides were deparaffinized and rehydrated in a declining graded sequence of ethanol (100%, 90%, and 70%) for immunostaining. A cooker was used to incubate the slides at high pressure after they were submerged in an antigen retrieval solution (S169984-2, Dako). To inhibit endogenous peroxidase activity, the slides were treated with 3% hydrogen peroxide for 30 minutes, after cooling on ice for 1 hour. After 2 PBS washes, the slides were incubated for 1 to 2 hours at room temperature in a humidity chamber with serum-free protein blocks (X0909; Dako). Mouse tissues were treated with mouse-on-mouse (vector) reagent before antibody application. Tissues were incubated with primary antibodies overnight at 4 °C. After 3 PBS washes, the specimens were incubated for 15 minutes at room temperature with either horseradish peroxidase–conjugated anti-mouse secondary antibodies (Dako) or horseradish peroxidase–conjugated anti-rabbit secondary antibodies (Dako). Mayer hematoxylin (Dako) was used for nuclear staining for IHC analysis, whereas diaminobenzidine was used for signal expression.

For IF, Texas Red dye (Invitrogen) and fluorescein isothiocyanate (Invitrogen)-, Cy3 (Invitrogen)-, and Cy5 (Invitrogen)-conjugated secondary antibodies were used to detect the primary antibodies derived from various species. 6-diamino-2-phenylindole (Sigma) was used for nuclear staining. LSM 770 (Zeiss) and EVOS-M5000 (Thermo Fisher Scientific) microscopes were used for confocal and IF imaging, respectively.

RNAscope probes specific for *H pylori* (Probe-B-H. pylori-16S, Cat. No. 542931) and *H felis* (Probe-B- H. felis-omp2-C1, Cat. No. 1281251-C1), along with the RNAscope 2.5 HD Detection Kit, were purchased from Advanced Cell Diagnostics and used according to the manufacturer's instructions for ISH. A ready-to-use protease, hydrogen peroxide, and a target retrieval solution were used as pretreatments for the deparaffinized formalin-fixed paraffin-embedded slides. Prewarmed probes were placed on slides and incubated for 2 hours at 40 °C in a HybEZ oven (Advanced Cell Diagnostics). After the signal amplification, rapid red dye development was performed. Counterstaining was performed using Mayer's hematoxylin.

For ISH quantification, gland-base colonization was calculated as the ratio of ISH-positive glands to total glands per field. SPEM was quantified as the percentage of GSII–GIF double-positive cells among GIF-positive chief cells, whereas CD44v9- and TFF2-positive cells were counted per gland. The proliferative zone was evaluated by Ki67 immunostaining based on the distribution of Ki67-positive cells along the gland axis.

## Scanning Electron Microscopy

Two-D organoid samples were fixed in Karnovsky's fixative (2% glutaraldehyde and 2% paraformaldehyde in 0.1 M phosphate buffer, pH 7.4) for 24 hours. Samples were then washed twice for 30 minutes with 0.1 M phosphate buffer and postfixed with 1% osmium tetroxide ($OsO_4$) for 2 hours. Dehydration was performed using a graded ethanol series (50%–100%). The samples were dried using a LEICA EM CPD300 critical point dryer and coated with platinum using a LEICA EM ACE600 ion-sputter coater. Imaging was conducted using a field-emission scanning electron microscope (MERLIN, ZEISS).

## Transmission Electron Microscopy

Two-D organoids were fixed under the same conditions for 12 hours and postfixed with 1% $OsO_4$ for 2 hours. The samples were dehydrated in a graded ethanol series (50%–100%) and treated with propylene oxide for 10 minutes at each step. The specimens were embedded using a Poly/Bed 812 kit (Polysciences) and polymerized at 65 °C for 12 hours in an electron microscope oven (TD-700, DOSAKA). Semithin sections (~200 nm) were prepared using a diamond knife and stained with toluidine blue for light microscopy. Areas of interest were then sectioned into ultrathin sections (~80 nm), mounted on copper grids, and sequentially stained with 3% uranyl acetate for 30 minutes and 3% lead citrate for 7 minutes. Imaging was performed using a JEM-1011 transmission electron microscope (JEOL) equipped with a Megaview III CCD camera (Soft Imaging System) at an accelerating voltage of 80 kV.

## RNA Sequencing and Transcriptomic Analysis

RNA-seq libraries were prepared from AGS cells using the QuantSeq 3′ mRNA-Seq Library Prep Kit (Lexogen) and sequenced on an Illumina platform. Reads were aligned to the human reference genome (GRCh38) using STAR aligner. DEGs were identified using independent *t* tests and fold-change criteria. GO enrichment was assessed through the GO database (www.geneontology.org), and GSEA (GSEA v4.0.3, Broad Institute) was performed. Data analysis and visualization of DEGs were performed using ExDEGA (Ebiogen). RNA-seq data have been deposited in the Gene Expression Omnibus (accession no. GSE275727).

## Single-Cell RNA Sequencing Analysis

To analyze the expression levels in human gastric cancer, public single-cell RNA sequencing data (GSE150290) were used to reanalyze samples from normal tissues of 5 patients without cancer and paired cancerous and adjacent tissues from 24 patients with gastric cancer. In summary, a gene-count matrix was constructed from the fastq files

mapped to the GRCh38 reference genome using Cell Ranger software (v7.0.0) provided by 10× Genomics. Following a previous approach, low-quality cells were filtered based on the following criteria: RNA counts >20,000, RNA feature counts <200 or >2000, and mitochondrial gene and hemoglobin gene percentages >30% and 10%, respectively. The remaining cells were normalized and clustered using the unsupervised clustering method with the FindClusters function (resolution = 0.3) in the Seurat R package (v5.0.0). Clusters were annotated by manually inspecting DEGs compared with previously defined cell type markers. Single-cell analysis consisted of GSE150290 data previously deposited by our research team in the Gene Expression Omnibus database. Single-cell quality control and data preprocessing were performed in the same manner as described in our previous study.[39]

### Quantitative Reverse-Transcription Polymerase Chain Reaction

Total RNA was isolated using QIAzol reagent according to the manufacturer's instructions. Complementary DNA was synthesized from 1 μg of RNA using reverse transcriptase, and RT-PCR was performed using SYBR Green master mix on a real-time PCR system. Glyceraldehyde-3-phosphate dehydrogenase (GAPDH) was used as the housekeeping control.

*OTU deubiquitinase, ubiquitin aldehyde binding 2.* Forward: 5′-CCTGCTTTTGACTGGGTTCC-3′
Reverse: 5′-CTCATGGTTCCCCTGACACT-3′

*Yes-associated protein.* Primer set 1
Forward: 5′-AGGGAGGAAGGAAGGAACAA-3′
Reverse: 5′-GAGAAACAGCTCCCAACTGC-3′

*Caudal type homeobox 2.* Forward: 5′-GACGTGAGCATGTACCCTAGC-3′
Reverse: 5′-GCGTAGCCATTCCAGTCCT-3′

*Zonula occludens-1.* Forward: 5′-AGAAGATAGCCCTGCAGC-3′
Reverse: 5′-AGTCCATAGGGAGATTCC-3′

*Glyceraldehyde-3-phosphate dehydrogenase (housekeeping gene).* Forward: 5′-CGACCACTTTGTCAAGCTCA-3′
Reverse: 5′-AGGGGTCTACATGGCAACTG-3′

### Antibodies

The following primary antibodies were used for IHC, IF, or WB analysis: YAP (Cell Signaling, 4912S, RRID:AB_2218911), TAZ (BD, 560,235, RRID:AB_1645338), YAP/TAZ (Cell Signaling, 8418S, RRID:AB_10950494), LATS2 (Cell Signaling, 5888S, RRID:AB_10835233), phospho-YAP (Ser127) (Cell Signaling, 4911, RRID:AB_2218913), SUMO2/3 (Cell Signaling, 4971, RRID:AB_2198425), CDX2 (Abcam, ab76541, RRID:AB_1523334), TFF2 (Invitrogen, PA5-57781, RRID:AB_2648410), MUC5AC (Abcam, ab77576, RRID:AB_2146994), MUC6 (Abcam, ab212646, RRID:AB_2864391), CD44v9 (CosmoBio, CAC-LKG-M002, RRID:AB_2910608), E-cadherin (Abcam, ab40772, RRID:AB_731493), Mist1 (Santa Cruz, sc-80984, RRID:AB_2065216), PGC (Novus, NBP1-91011, RRID:AB_11023080), ZO-1 (Invitrogen, 339,100, RRID:AB_87181), OTUB2 (ATLAS, HPA002329, RRID:AB_1079537; Invitrogen, PA5-99680, RRID:AB_2818613), CagA (Santa Cruz, sc-28368, RRID:AB_628229), *H pylori* (Novus, NBP2-29479, RRID:AB_3276838), ubiquitin (Santa Cruz, sc-8017, RRID:AB_2762364), and Lamin B1 (Santa Cruz, sc-374015, RRID:AB_10947408).

### Statistical Analysis

All experiments were performed at least in triplicate. Data are presented as mean ± standard error of the mean. Statistical significance was determined using 2-tailed unpaired Student's $t$ tests or 1-/2-way analysis of variance (ANOVA) with Tukey's post hoc test (GraphPad Prism v10). A $P$ value < .05 indicated statistical significance. Statistical significance is denoted by asterisks as follows: *$P$ < .05; **$P$ < .01; ***$P$ < .001; and ****$P$ < .0001. All authors had access to the study data and had reviewed and approved the final manuscript.

## Supplementary Material

Note: To access the supplementary material accompanying this article, visit the full text version at https://doi.org/10.1016/j.jcmgh.2026.101814.

## References


1. Toh JWT, Wilson RB. Pathways of gastric carcinogenesis, Helicobacter pylori virulence and interactions with antioxidant systems, vitamin C and phytochemicals. Int J Mol Sci 2020;21:6451.
2. Thrift AP, Wenker TN, El-Serag HB. Global burden of gastric cancer: epidemiological trends, risk factors, screening and prevention. Nat Rev Clin Oncol 2023;20:338–349.
3. Park YH, Kim N. Review of atrophic gastritis and intestinal metaplasia as a premalignant lesion of gastric cancer. J Cancer Prev 2015;20:25–40.
4. Lee SD, Jeong H, Hwang BR, et al. Helicobacter pylori promotes epithelial-to-mesenchymal transition by downregulating CK2β in gastric cancer cells. Biochim Biophys Acta Mol Basis Dis 2023;1869:166588.
5. Yu BM, Lee SD, Hwang BR, et al. Application of an organoid-based model to explore Helicobacter pylori–human gastric epithelium interaction in vitro. Front Cell Infect Microbiol 2025;15:1572244.
6. Takahashi-Kanemitsu A, Knight CT, Hatakeyama M. Molecular anatomy and pathogenic actions of Helicobacter pylori CagA that underpin gastric carcinogenesis. Cell Mol Immunol 2020;17:50–63.
7. Noto JM, Peek RM Jr. The Helicobacter pylori cag Pathogenicity Island. Methods Mol Biol 2012;921:41–50.
8. Amieva MR, Vogelmann R, Covacci A, et al. Disruption of the epithelial apical-junctional complex by Helicobacter pylori CagA. Science 2003;300:1430–1434.
9. Caron TJ, Scott KE, Fox JG, Hagen SJ. Tight junction disruption: Helicobacter pylori and dysregulation of the gastric mucosal barrier. World J Gastroenterol 2015;21:11411–11427.
10. Alzahrani S, Lina TT, Gonzalez J, et al. Effect of Helicobacter pylori on gastric epithelial cells. World J Gastroenterol 2014;20:12767–12780.

11. Contreras-Panta EW, Lee SH, Won Y, et al. Interleukin 13 promotes maturation and proliferation in metaplastic gastroids. Cell Mol Gastroenterol Hepatol 2024;18:101366.
12. Messina B, Lo Sardo F, Scalera S, et al. Hippo pathway dysregulation in gastric cancer: from Helicobacter pylori infection to tumor promotion and progression. Cell Death Dis 2023;14:21.
13. Seeneevassen L, Dubus P, Gronnier C, Varon C. Hippo in gastric cancer: from signalling to therapy. Cancers 2022;14:2282.
14. Pobbati AV, Kumar R, Rubin BP, Hong W. Therapeutic targeting of TEAD transcription factors in cancer. Trends Biochem Sci 2023;48:450–462.
15. Zhou GX, Li XY, Zhang Q, et al. Effects of the hippo signaling pathway in human gastric cancer. Asian Pac J Cancer Prev 2013;14:5199–5205.
16. Zhao W, Wang M, Cai M, et al. Transcriptional co-activators YAP/TAZ: Potential therapeutic targets for metastatic breast cancer. Biomed Pharmacother 2021;133:110956.
17. Papavassiliou KA, Sofianidi AA, Papavassiliou AG. YAP/TAZ-TEAD signalling axis: A new therapeutic target in malignant pleural mesothelioma. J Cell Mol Med 2024;28:e18330.
18. Li N, Xu X, Zhan Y, et al. YAP and $\beta$-catenin cooperate to drive H. pylori-induced gastric tumorigenesis. Gut Microbes 2023;15:2192501.
19. Molina-Castro SE, Tiffon C, Giraud J, et al. The hippo kinase LATS2 controls Helicobacter pylori-induced epithelial-mesenchymal transition and intestinal metaplasia in gastric mucosa. Cell Mol Gastroenterol Hepatol 2020;9:257–276.
20. Li N, Feng Y, Hu Y, et al. Helicobacter pylori CagA promotes epithelial mesenchymal transition in gastric carcinogenesis via triggering oncogenic YAP pathway. J Exp Clin Cancer Res 2018;37:280.
21. Fischer AS, Müllerke S, Arnold A, et al. R-spondin/YAP axis promotes gastric oxyntic gland regeneration and Helicobacter pylori-associated metaplasia in mice. J Clin Invest 2022;132:e151363.
22. Loe AKH, Rao-Bhatia A, Wei Z, et al. YAP targetome reveals activation of SPEM in gastric pre-neoplastic progression and regeneration. Cell Rep 2023;42:113497.
23. Goldenring JR, Mills JC. Cellular plasticity, reprogramming, and regeneration: metaplasia in the stomach and beyond. Gastroenterology 2022;162:415–430.
24. Zhang Z, Du J, Wang S, et al. OTUB2 promotes cancer metastasis via hippo-independent activation of YAP and TAZ. Mol Cell 2019;73:7–21.e27.
25. Li J, Zhang N, Li M, et al. The emerging role of OTUB2 in diseases: from cell signaling pathway to physiological function. Front Cell Dev Biol 2022;10:820781.
26. Ren W, Xu Z, Chang Y, et al. Pharmaceutical targeting of OTUB2 sensitizes tumors to cytotoxic T cells via degradation of PD-L1. Nat Commun 2024;15:9.
27. Sharma R, Sharma S, Shriwas P, et al. Intra-tumoral YAP and TAZ heterogeneity drives collective NSCLC invasion that is targeted by SUMOylation inhibitor TAK-981. iScience 2024;27:111133.
28. He J, Hu W, Ouyang Q, et al. Helicobacter pylori infection induces stem cell-like properties in Correa cascade of gastric cancer. Cancer Lett 2022;542:215764.
29. Bartfeld S, Bayram T, van de Wetering M, et al. In vitro expansion of human gastric epithelial stem cells and their responses to bacterial infection. Gastroenterology 2015;148:126–136.e126.
30. Boccellato F, Woelffling S, Imai-Matsushima A, et al. Polarised epithelial monolayers of the gastric mucosa reveal insights into mucosal homeostasis and defence against infection. Gut 2019;68:400–413.
31. Joo S, Fang S. Bile acids and gastric intestinal metaplasia: exploring a new feedback loop. Gut Liver 2024;18:365–367.
32. Koide T, Koyanagi-Aoi M, Uehara K, et al. CDX2-induced intestinal metaplasia in human gastric organoids derived from induced pluripotent stem cells. iScience 2022;25:104314.
33. Chong Y, Yu D, Lu Z, Nie F. Role and research progress of spasmolytic polypeptide-expressing metaplasia in gastric cancer (Review). Int J Oncol 2024;64:33.
34. Petersen CP, Mills JC, Goldenring JR. Murine models of gastric corpus preneoplasia. Cell Mol Gastroenterol Hepatol 2017;3:11–26.
35. Nam KT, Lee HJ, Sousa JF, et al. Mature chief cells are cryptic progenitors for metaplasia in the stomach. Gastroenterology 2010;139:2028–2037.e2029.
36. Mills JC, Goldenring JR. Metaplasia in the stomach arises from gastric chief cells. Cell Mol Gastroenterol Hepatol 2017;4:85–88.
37. Radyk MD, Burclaff J, Willet SG, Mills JC. Metaplastic cells in the stomach arise, independently of stem cells, via dedifferentiation or transdifferentiation of chief cells. Gastroenterology 2018;154:839–843.e832.
38. Bockerstett KA, Lewis SA, Noto CN, et al. Single-cell transcriptional analyses identify lineage-specific epithelial responses to inflammation and metaplastic development in the gastric corpus. Gastroenterology 2020;159:2116–2129.e2114.
39. Kim J, Park C, Kim KH, et al. Single-cell analysis of gastric pre-cancerous and cancer lesions reveals cell lineage diversity and intratumoral heterogeneity. NPJ Precis Oncol 2022;6:9.
40. Flotho A, Melchior F. Sumoylation: a regulatory protein modification in health and disease. Annu Rev Biochem 2013;82:357–385.
41. Gareau JR, Lima CD. The SUMO pathway: emerging mechanisms that shape specificity, conjugation and recognition. Nat Rev Mol Cell Biol 2010;11:861–871.
42. Song X, Chen HX, Wang XY, et al. H. pylori-encoded CagA disrupts tight junctions and induces invasiveness of AGS gastric carcinoma cells via Cdx2-dependent targeting of Claudin-2. Cell Immunol 2013;286:22–30.
43. Jeong H, Lee B, Kim KH, et al. WFDC2 promotes spasmolytic polypeptide-expressing metaplasia through the up-regulation of IL33 in response to injury. Gastroenterology 2021;161:953–967.e915.

44. Geahlen JH, Lapid C, Thorell K, et al. Evolution of the human gastrokine locus and confounding factors regarding the pseudogenicity of GKN3. Physiol Genomics 2013;45:667–683.
45. Druffner SR, Venkateshwaraprabu S, Khadka S, et al. Comparison of gastric inflammation and metaplasia induced by Helicobacter pylori or Helicobacter felis colonization in mice. Microbiol Spectr 2024;12:e0001524.
46. Prinz C, Hafsi N, Voland P. Helicobacter pylori virulence factors and the host immune response: implications for therapeutic vaccination. Trends Microbiol 2003;11:134–138.
47. Ansari S, Yamaoka Y. Animal models and Helicobacter pylori infection. J Clin Med 2022;11:3141.
48. Lee JH, Kim S, Han S, et al. p57(Kip2) imposes the reserve stem cell state of gastric chief cells. Cell Stem Cell 2022;29:826–839.e829.
49. Willet SG, Lewis MA, Miao ZF, et al. Regenerative proliferation of differentiated cells by mTORC1-dependent paligenosis. EMBO J 2018;37:e98311.
50. Brown JW, Cho CJ, Mills JC. Paligenosis: cellular remodeling during tissue repair. Annu Rev Physiol 2022; 84:461–483.
51. Mills JC, Stanger BZ, Sander M. Nomenclature for cellular plasticity: are the terms as plastic as the cells themselves? EMBO J 2019;38:e103148.

**Received October 21, 2025. Accepted May 8, 2026.**

**Correspondence**
Address correspondence to: Yong Chan Lee, MD, PhD, Department of Internal Medicine, Institute of Gastroenterology, Yonsei University College of Medicine, 250 Seongsanno, Seodaemun-gu, Seoul 120-752, Republic of Korea. e-mail: leeyc@yuhs.ac; or Ki Taek Nam, DVM, PhD, Severance Biomedical Science Institute, Yonsei University College of Medicine, 50-1 Yonsei-ro, Seodaemun-gu, Seoul 03722, Republic of Korea. e-mail: kitaek@yuhs.ac.

**Acknowledgments**
The authors thank Haengdeung Jeong, Jiseon Kim, and Seok Young Hwang for technical assistance.

**CRediT Authorship Contributions**
Byeong Min Yu, PhD (Formal analysis: Lead; Methodology: Lead; Writing – original draft: Lead)
So Dam Lee, PhD (Data curation: Supporting; Formal analysis: Supporting; Methodology: Supporting)
Bo Ram Hwang, PhD Candidate (Investigation: Supporting; Methodology: Supporting)
Haengdeung Jeong, PhD (Methodology: Supporting; Resources: Supporting)
Jiseon Kim, PhD Candidate (Methodology: Supporting; Resources: Supporting)
Seokyoung Hwang, PhD Candidate (Methodology: Supporting)
Minsoo Noh, PhD (Methodology: Supporting)
Ki Taek Nam, DVM, PhD (Conceptualization: Lead; Investigation: Lead; Methodology: Lead; Resources: Lead)
Yong Chan Lee, MD, PhD (Conceptualization: Lead; Funding acquisition: Lead; Investigation: Lead; Supervision: Lead; Writing – review & editing: Lead)

**Conflicts of interest**
The authors disclose no conflicts.

**Funding**
This study was supported by the National Research Foundation of Korea (NRF) and the Yonsei University College of Medicine Faculty Research Grant, awarded as follows: Yong Chan Lee: NRF (2022R1A2C209190213); Yonsei University College of Medicine Faculty Research Grant [6-2022-0132]; Ki Taek Nam: NRF (2022R1A2C3007850); and Byeong Min Yu: NRF (2022R1A6A3A1306658012). The funders had no role in study design, data collection and analysis, decision to publish, or preparation of the manuscript.

**Data Availability**
Bulk QuantSeq 3′ mRNA-seq data have been deposited in Gene Expression Omnibus under accession GSE275727. Single-cell analysis was based on GSE150290 data previously deposited by our research team in the Gene Expression Omnibus database.

Cellular and Molecular Gastroenterology and Hepatology, Volume *20*

# Supplemental information

## Yes-Associated Protein Drives *Helicobacter pylori*–Induced Metaplastic Changes in Gastric Epithelium

**Byeong Min Yu, So Dam Lee, Bo Ram Hwang, Haengdueng Jeong, Jiseon Kim, Seokyoung Hwang, Minsoo Noh, Ki Taek Nam, and Yong Chan Lee**

Figure 1

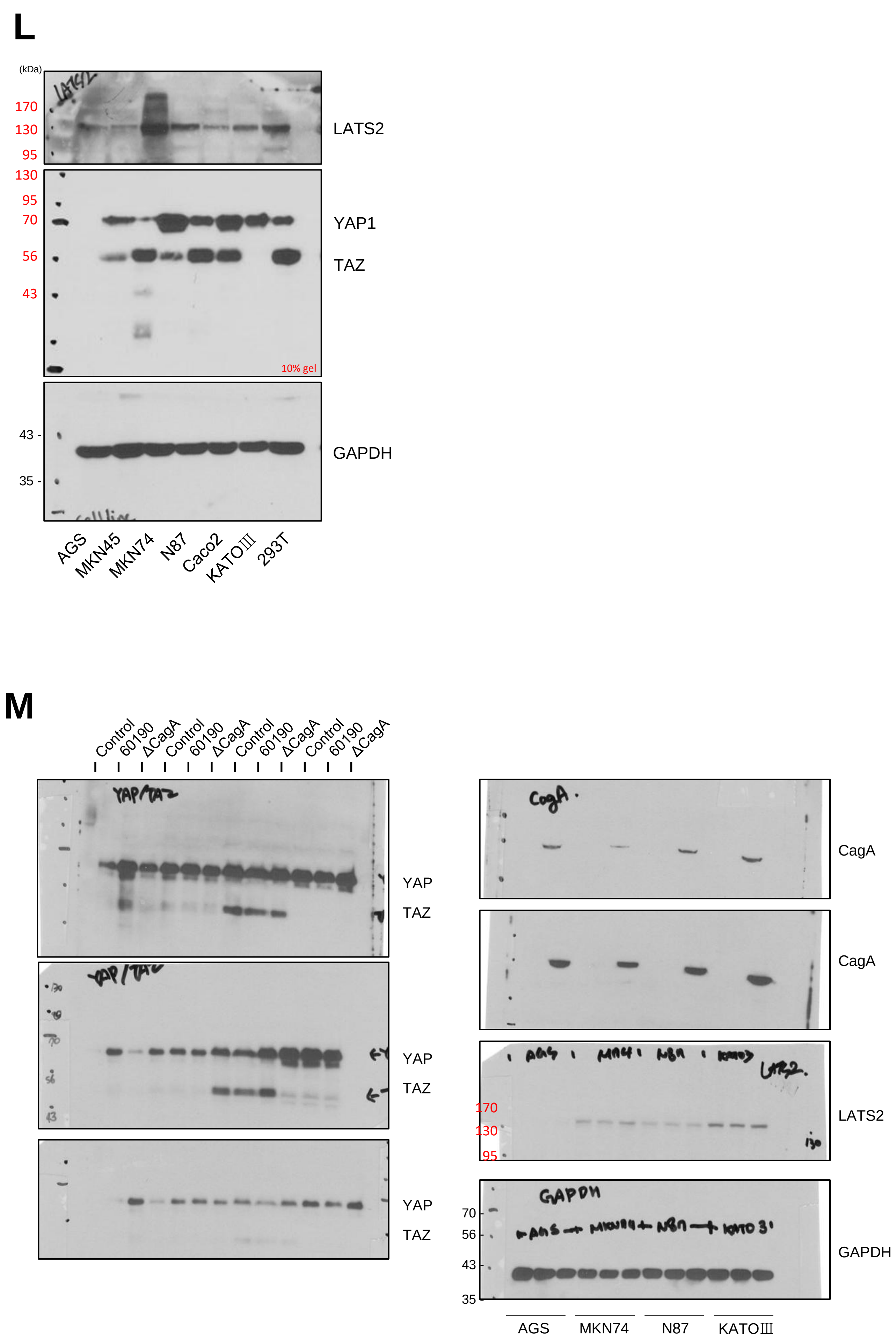
L
(kDa)
170
130
95
LATS2
130
95
70
56
43
YAP1
TAZ
10% gel
43 -
35 -
GAPDH
AGS
MKN45
MKN74
N87
Caco2
KATOIII
293T
M
Control
60190
ΔCagA
Control
60190
ΔCagA
Control
60190
ΔCagA
Control
60190
ΔCagA
YAP
TAZ
YAP
TAZ
YAP
TAZ
CagA
CagA
170
130
95
LATS2
70
56
43
35
GAPDH
AGS
MKN74
N87
KATOIII

# Figure 2

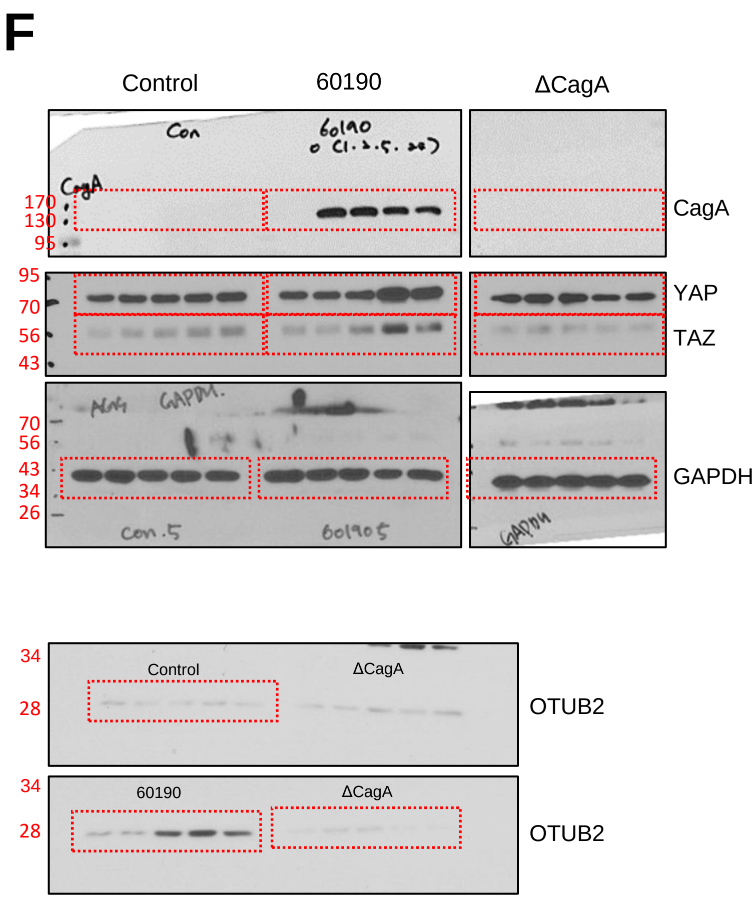

F
Control
60190
ΔCagA
CagA
YAP
TAZ
GAPDH
Control
ΔCagA
OTUB2
60190
ΔCagA
OTUB2
170
130
95
70
56
43
34
26
28


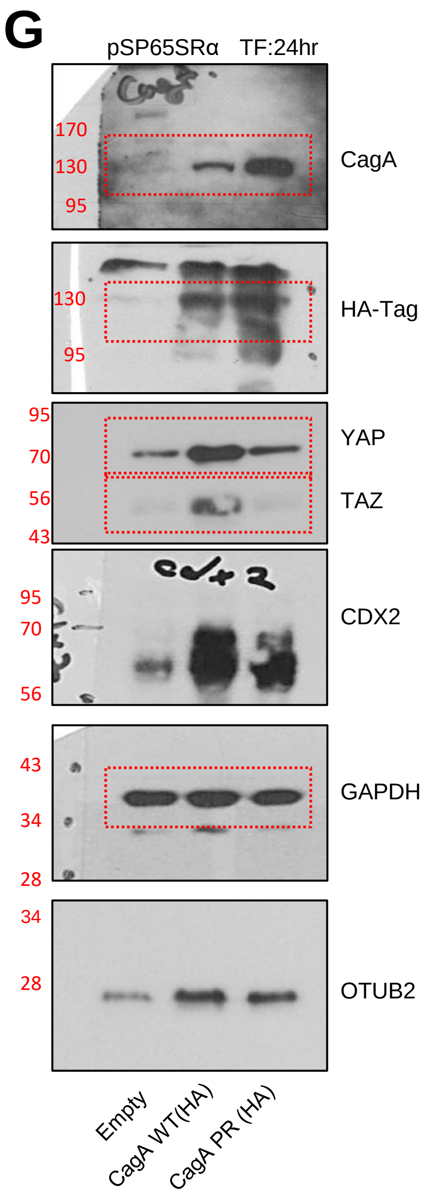

G
pSP65SRα
TF:24hr
CagA
HA-Tag
YAP
TAZ
CDX2
GAPDH
OTUB2
Empty
CagA WT(HA)
CagA PR (HA)
170
130
95
70
56
43
34
28

# Figure 3

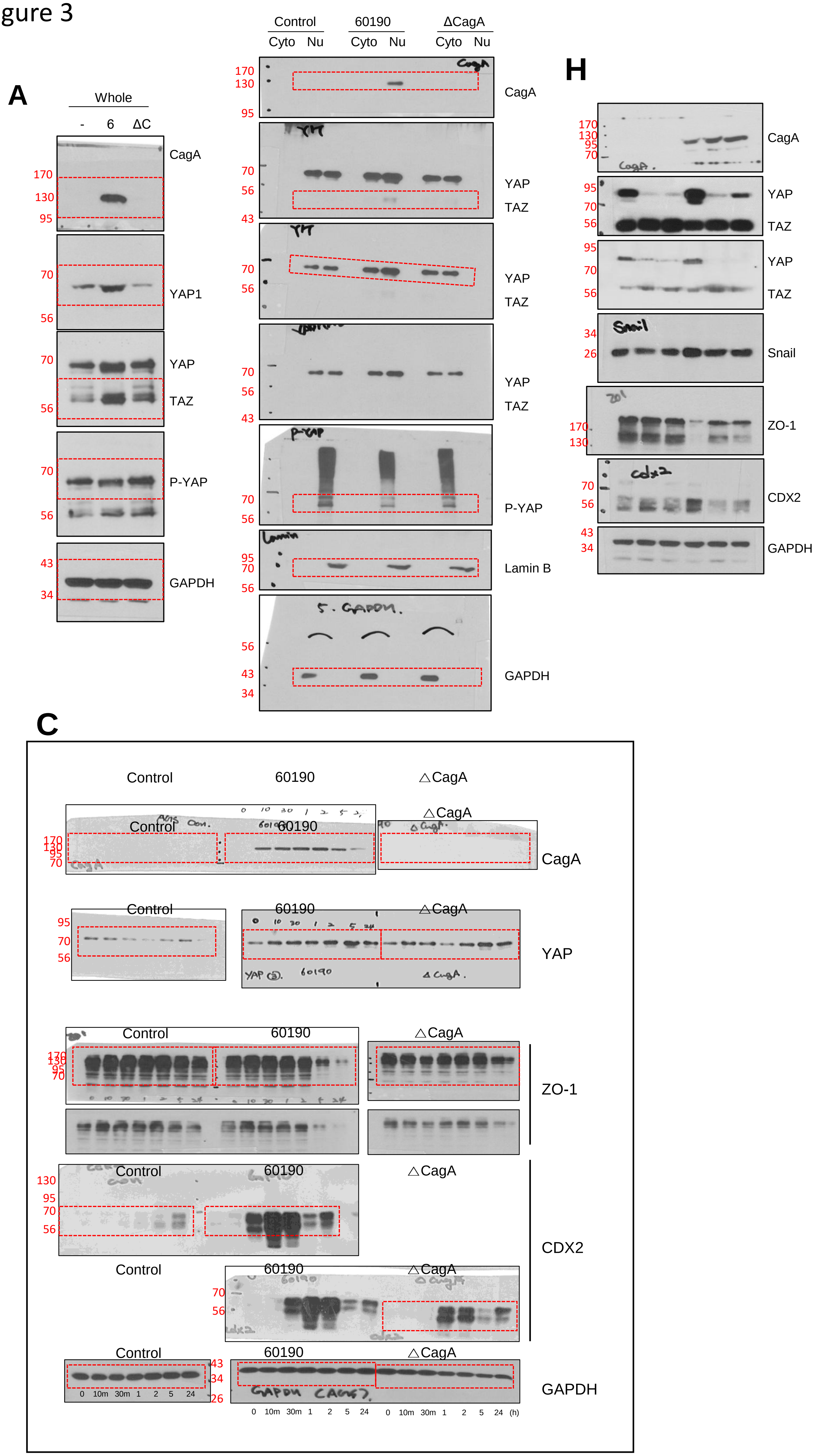
A
Whole
- 6 ΔC
CagA
YAP1
YAP
TAZ
P-YAP
GAPDH
Control 60190 ΔCagA
Cyto Nu Cyto Nu Cyto Nu
CagA
YAP
TAZ
P-YAP
Lamin B
GAPDH
H
CagA
YAP
TAZ
Snail
ZO-1
CDX2
GAPDH
C
Control
60190
△CagA
CagA
YAP
ZO-1
CDX2
GAPDH
0 10m 30m 1 2 5 24
0 10m 30m 1 2 5 24 0 10m 30m 1 2 5 24 (h)

# Figure 4

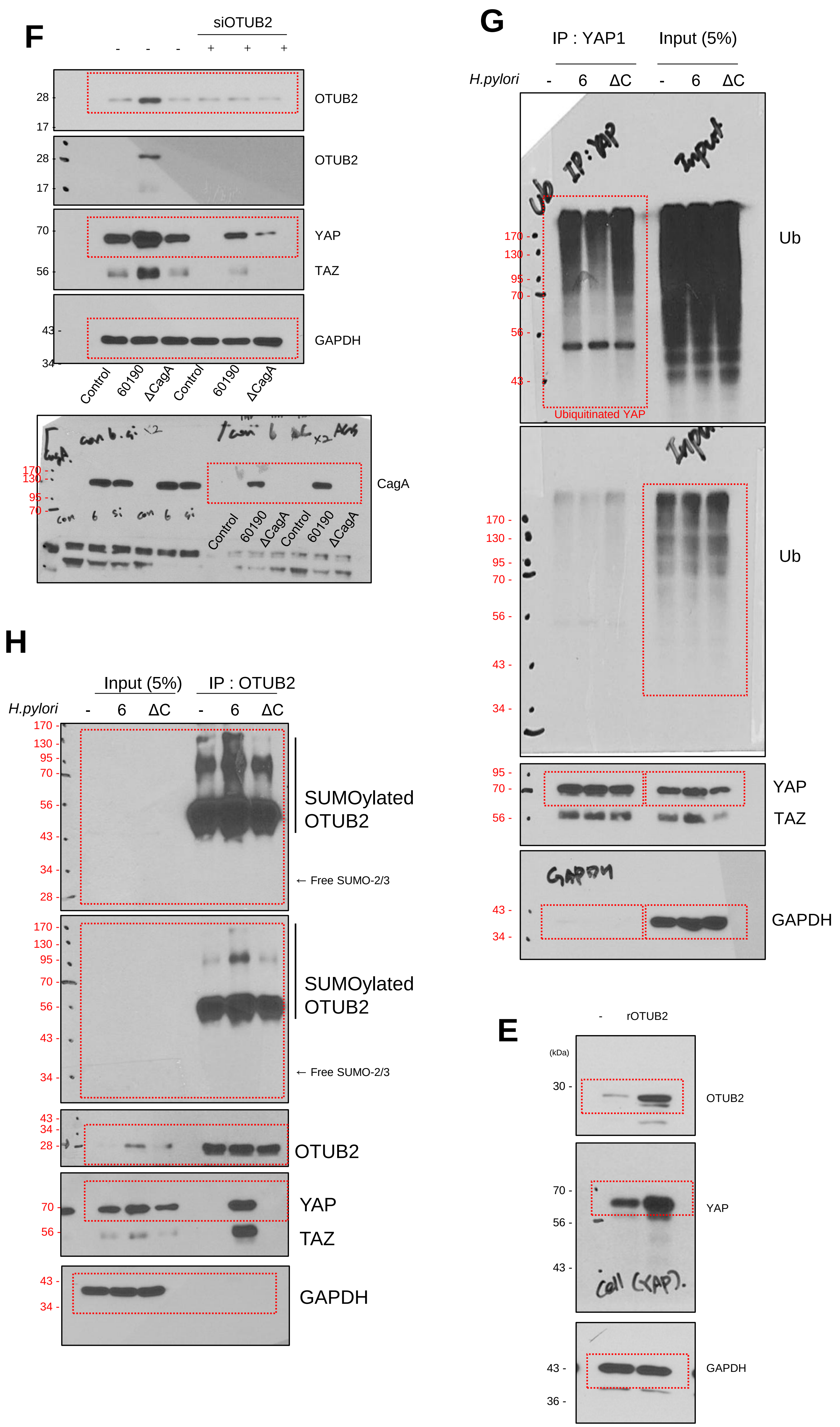

F
siOTUB2
- - - + + +
28
17
OTUB2
28
17
OTUB2
70
56
YAP
TAZ
43 -
34 -
GAPDH
Control
60190
ΔCagA
Control
60190
ΔCagA
170
130
95
70
CagA
Control
60190
ΔCagA
Control
60190
ΔCagA
G
IP : YAP1
Input (5%)
H.pylori
- 6 ΔC - 6 ΔC
170 -
130 -
95 -
70 -
56 -
43 -
Ubiquitinated YAP
Ub
170 -
130 -
95 -
70 -
56 -
43 -
34 -
Ub
95 -
70 -
56 -
YAP
TAZ
43 -
34 -
GAPDH
H
Input (5%)
IP : OTUB2
H.pylori
- 6 ΔC - 6 ΔC
170 -
130 -
95 -
70 -
56 -
43 -
34 -
28 -
SUMOylated OTUB2
← Free SUMO-2/3
170 -
130 -
95 -
70 -
56 -
43 -
34 -
SUMOylated OTUB2
← Free SUMO-2/3
43 -
34 -
28 -
OTUB2
70
56
YAP
TAZ
43 -
34 -
GAPDH
E
- rOTUB2
(kDa)
30 -
OTUB2
70 -
56 -
43 -
YAP
43 -
36 -
GAPDH

# Figure 4

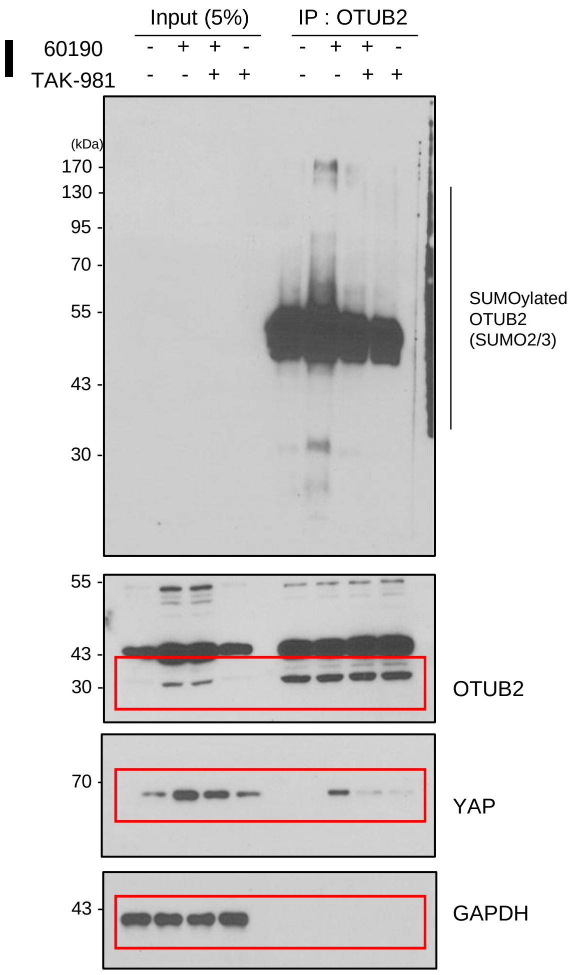
I
Input (5%)
IP : OTUB2
60190
TAK-981
(kDa)
170
130
95
70
55
43
30
SUMOylated
OTUB2
(SUMO2/3)
OTUB2
YAP
GAPDH
